# Divergent Evolution of *Legionella* RCC1 Repeat Effectors Defines the Range of Ran GTPase Cycle Targets

**DOI:** 10.1128/mBio.00405-20

**Published:** 2020-03-24

**Authors:** A. Leoni Swart, Bernhard Steiner, Laura Gomez-Valero, Sabina Schütz, Mandy Hannemann, Petra Janning, Michael Irminger, Eva Rothmeier, Carmen Buchrieser, Aymelt Itzen, Vikram Govind Panse, Hubert Hilbi

**Affiliations:** a Institute of Medical Microbiology, University of Zurich, Zurich, Switzerland; b Institut Pasteur, Unité de Biologie des Bactéries Intracellulaires, Paris, France; c CNRS UMR 3525, Paris, France; d Center for Integrated Protein Science Munich, Department of Chemistry, Technical University Munich, Garching, Germany; e Max Planck Institut für Molekulare Physiologie, Dortmund, Germany; f Max von Pettenkofer Institute, Ludwig-Maximilians University Munich, Munich, Germany; g Institute for Biochemistry and Signal Transduction, University Medical Center Hamburg-Eppendorf, Hamburg, Germany; Yale University School of Medicine

**Keywords:** amoeba, bacterial evolution, *Acanthamoeba*, *Dictyostelium*, effector protein, guanine nucleotide exchange factor, host-pathogen interaction, *Legionella*, macrophage, microtubule, pathogen vacuole, phosphoinositide lipid, small GTPase, type IV secretion, vesicle trafficking

## Abstract

Legionella pneumophila governs its interactions with host cells by secreting >300 different “effector” proteins. Some of these effectors contain eukaryotic domains such as the RCC1 (regulator of chromosome condensation 1) repeats promoting the activation of the small GTPase Ran. In this report, we reveal a conserved pattern of L. pneumophila RCC1 repeat genes, which are distributed in two main clusters of strains. Accordingly, strain Philadelphia-1 contains two RCC1 genes implicated in bacterial virulence, *legG1* (*Legionella* eukaryotic gene 1), and *ppgA*, while strain Paris contains only one, *pieG*. The RCC1 repeat effectors localize to different cellular compartments and bind distinct components of the Ran GTPase cycle, including Ran modulators and the small GTPase itself, and yet they all promote the activation of Ran. The *pieG* gene spans the corresponding open reading frames of *legG1* and a separate adjacent upstream gene, *lpg1975*. *legG1* and *lpg1975* are fused upon addition of a single nucleotide to encode a protein that adopts the binding specificity of PieG. Thus, a point mutation in *pieG* splits the gene, altering the effector target. These results indicate that divergent evolution of RCC1 repeat effectors defines the Ran GTPase cycle targets and that modulation of different components of the cycle might fine-tune Ran activation during *Legionella* infection.

## Abstract (precis)

Legionella pneumophila is a ubiquitous environmental bacterium which, upon inhalation, causes a life-threatening pneumonia termed Legionnaires’ disease. The opportunistic pathogen grows in amoebae and macrophages by employing a “type IV” secretion system, which secretes more than 300 different “effector” proteins into the host cell, where they subvert pivotal processes. The function of many of these effector proteins is unknown, and their evolution has not been studied. L. pneumophila RCC1 repeat effectors target the small GTPase Ran, a molecular switch implicated in different cellular processes such as nucleocytoplasmic transport and microtubule cytoskeleton dynamics. We provide evidence that one or more RCC1 repeat genes are distributed in two main clusters of L. pneumophila strains and have divergently evolved to target different components of the Ran GTPase activation cycle at different subcellular sites. Thus, L. pneumophila employs a sophisticated strategy to subvert host cell Ran GTPase during infection.

## INTRODUCTION

During coevolution with eukaryotic host cells, intracellular bacterial pathogens developed large repertoires of translocated “effector” proteins, which modulate host organelles and processes in sophisticated manners (1, 2). Legionella pneumophila is an amoeba-resistant environmental bacterium that upon inhalation causes a pneumonia called Legionnaires’ disease (3, 4). The opportunistic pathogen injects more than 300 different putative effector proteins through the Icm/Dot (intracellular multiplication/defective organelle trafficking) type IV secretion system (T4SS) into host cells (1, 5–7). The Icm/Dot-translocated proteins determine the infection process by subverting signal transduction, cytoskeleton dynamics, and membrane trafficking. Specifically, the effector proteins govern the formation of the pathogen’s intracellular replication niche, termed the *Legionella*-containing vacuole (LCV) (8–10). A crucial feature of LCVs is the phosphoinositide (PI) lipid conversion from phosphatidylinositol 3-phosphate (PtdIns(3)*P*) to PtdIns(4)*P* (11–14). Among the plethora of effectors, only a few have been functionally characterized, and these target PI lipids (12, 15–19), sphingolipid metabolism (20), small GTPases (5, 21–25), the ubiquitination machinery (7), trafficking complexes (26–30), protein translation (31–33), or gene transcription (34, 35).

Proteomics analyses of intact LCVs purified from either Dictyostelium discoideum amoebae or macrophages indicated that Ran, Ran binding protein 1 (RanBP1), RanBP2, RanGAP1 (Ran GTPase-activating protein 1), RCC1 (regulator of chromosome condensation 1), and RCC2 might be host components implicated in pathogen vacuole formation (36–38). The small GTPase Ran has pleiotropic functions in different compartments of eukaryotic cells (39, 40). These include nucleocytoplasmic transport (41), mitotic spindle assembly and postmitotic nuclear envelope formation (42, 43), as well as endocytic receptor trafficking (44) and the modulation of cytoplasmic (noncentrosomal) microtubule dynamics (45). Ran is activated by the guanine nucleotide exchange factor (GEF) RCC1 (regulator of chromosome condensation 1), which facilitates the exchange of GDP with GTP (46). In turn, Ran(GTP) is inactivated by cytoplasmic RanGAP1 in concert with RanBP1, which specifically binds to activated Ran (39, 40). The presence of Ran and RanBP1 on LCVs during infection was validated by fluorescence microscopy, and both components of the Ran GTPase cycle were found to be implicated in intracellular replication of L. pneumophila by RNA interference (47).

The genome of L. pneumophila harbors a number of genes encoding proteins whose closest homologs are eukaryotic counterparts (48–50). Among these, *legG1* (*lpg1976*) (49), *ppgA* (*lpg2224*) (51), and *pieG* (*lpp1959*) (51, 52) encode effectors that contain RCC1 repeats predicted with high confidence on the basis of the amino acid sequences. LegG1 promotes Ran activation, microtubule stabilization, and LCV motility (47). Moreover, LegG1 stimulates the chemotactic migration of D. discoideum, macrophages and neutrophils, the motility of which is hyperinhibited by an L. pneumophila mutant strain lacking *legG1* (53). LegG1 (alias MitF) is also required for mitochondrion fission and concomitant inhibition of mitochondrial respiration during L. pneumophila infection (54). LegG1 and PieG contain a C-terminal CAAX motif, which is prenylated by the host prenylation machinery, thus facilitating membrane localization of the effector protein (52).

In this study, we employed bioinformatics and molecular analysis to assess the evolution and functions of L. pneumophila RCC1 repeat effectors. We show that RCC1 repeat effectors contribute to pathogen-host interactions and promote Ran activation as well as LCV and host cell motility by targeting different Ran GTPase cycle components. Accordingly, divergent evolution of RCC1 effectors defines the range of Ran GTPase cycle targets and might fine-tune the activation of the GTPase in a spatiotemporal manner during infection.

## RESULTS

### Distribution and structural comparison of L. pneumophila RCC1 repeat effectors.

**FIG 1:**
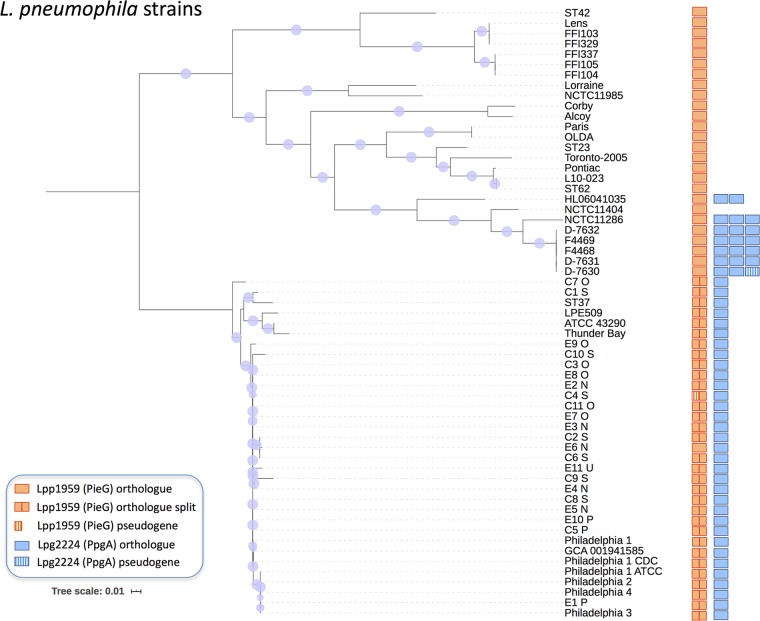
Distribution of L. pneumophila RCC1 repeat genes. The phylogenetic tree of 59 L. pneumophila strains was inferred using the fast core genome multialigner Parsnp (https://github.com/marbl/parsnp). Circles at nodes represent bootstrap support, and the size of each circle is proportional to the corresponding bootstrap value. The scale bar represents the estimated number of substitutions per site. For each strain, the presence or absence of the RCC1 repeat effector genes *lpp1959* (*pieG*) and *lpg2224* (*ppgA*) was assessed by OrthoMCL (https://orthomcl.org), and all of the strains were found to be distributed in two main clusters. In strain C4S, a split fragment of gene *lpp1959* is a pseudogene. Strain NCTC11404 does not harbor an *lpg2224* gene (verified by blast search), and one of three *lpg2224* copies in strain D-7630 is a pseudogene.

A bioinformatics analysis of *Legionella* genomes revealed that RCC1 repeat genes are present in many *Legionella* species (24 of 58 analyzed) and are conserved in all 59 L. pneumophila strains examined in this study (Fig. 1). While some of the L. pneumophila strains contain only one RCC1 repeat effector gene (e.g., strains Lens and Paris: *pieG*/*lpp1959*), others contain two single genes (e.g., strains Philadelphia-1 and C7O: *legG1*/*lpg1976* and *ppgA*/*lpg2224*) or, additionally, a duplicated *ppgA* gene. Indeed, the RCC1 repeat genes in L. pneumophila were found to be distributed in two main clusters, namely, the “Lens-Paris” cluster and the “Philadelphia-C7O” cluster, respectively. Most of the strains in the Lens-Paris cluster harbor only the *pieG* gene, the exceptions being a subcluster of seven strains containing *pieG* and two or three *ppgA* genes. The strains in the Philadelphia-C7O cluster are more closely related to each other and contain a split *pieG* gene (yielding *lpg1975 and legG1*; see below) as well as one *ppgA* gene. As an exception, strain E6N harbors single, intact *pieG* and *ppgA* genes.

The phylogenetic trees indicate that the RCC1 repeat genes have mostly followed the evolution of the strain (see Fig. S1 in the supplemental material). Strains E6N and NCTC11286 are exceptions, as they do not localize with the same strains in the phylogenetic trees of the strains and *lpp1959* genes (Fig. S1A). This suggests that horizontal gene transfer took place and explains why strain E6N groups with the strains where *lpp1959* is usually split. If present in two or more copies, *lpg2224* genes from the same strain do not group together (Fig. S1B). This finding suggests that one or several duplication events originated in a common ancestor, followed by posterior sequence divergence (and perhaps functional diversification) among the different duplications and posterior emergence of new strains from the common ancestor. Taken together, the data show that RCC1 repeat genes are conserved in L. pneumophila and occur with a distinct pattern comprising two different strain clusters.

10.1128/mBio.00405-20.2 FIG S1 Comparison of phylogenetic trees of L. pneumophila strains and RCC1 repeat effector genes. Download FIG S1, PDF file, 0.6 MB. Copyright © 2020 Swart et al. 2020 Swart et al. This content is distributed under the terms of the Creative Commons Attribution 4.0 International license.

The RCC1 repeat genes of L. pneumophila strain Philadelphia-1 and Paris contain 2 to 3 RCC1 repeats and encode the effectors PpgA (66 kDa) and LegG1/Lpg1976 (31 kDa), or PieG (53 kDa), respectively (Fig. S2). Previously, the acronyms “LegG1” and “PieG” were used synonymously (51). However, given the distinct features of the proteins (see below), here we use LegG1 and PieG exclusively for the orthologs of strains Philadelphia-1 and Paris, respectively. The positions of the predicted RCC1 repeats in the L. pneumophila effectors vary, but the level of homology with the RCC1 repeat of the human Ran GEF RCC1 is very high, and the homologous region includes the conserved GQLGLGE/D motif but lacks amino acids essential for GEF activity (Fig. S2). Moreover, structural predictions indicate that the L. pneumophila RCC1 repeat effectors adopt β-propeller structures like human RCC1 (55).

10.1128/mBio.00405-20.3 FIG S2 Structural comparison of eukaryotic RCC1 and L. pneumophila RCC1 repeat effector proteins. Download FIG S2, PDF file, 1.1 MB. Copyright © 2020 Swart et al. 2020 Swart et al. This content is distributed under the terms of the Creative Commons Attribution 4.0 International license.

### Role of L. pneumophila RCC1 repeat effectors in pathogen-host interactions.

To analyze the function of L. pneumophila RCC1 repeat effectors, strains lacking *ppgA* (LS03, Δ*ppgA*), *legG1* (Δ*legG1*), *ppgA* and *legG1* (LS01, Δ*ppgA*-Δ*legG1*), or *pieG* (LS08, Δ*pieG*) were constructed by deleting the genes from the chromosome by double homologous recombination (see Table S1A in the supplemental material). L. pneumophila Δ*ppgA*, Δ*legG1*, Δ*ppgA*-Δ*legG1* (strain Philadelphia-1), and Δ*pieG* (strain Paris) grew at the same rate as the parental strains in broth (Fig. S3A and B). Moreover, the overproduction of PieG (Paris) in strain Philadelphia-1 or of PpgA (Philadelphia-1) in strain Paris did not impair growth in broth (Fig. S3C and D).

10.1128/mBio.00405-20.4 FIG S3 Growth and competition of L. pneumophila strains lacking RCC1 repeat genes. Download FIG S3, PDF file, 0.7 MB. Copyright © 2020 Swart et al. 2020 Swart et al. This content is distributed under the terms of the Creative Commons Attribution 4.0 International license.

10.1128/mBio.00405-20.9 TABLE S1 (A) Bacterial strains and plasmids used in this study. (B) Oligonucleotides used in this study. (C) Oligonucleotides used for RNA interference. Download Table S1, PDF file, 0.2 MB. Copyright © 2020 Swart et al. 2020 Swart et al. This content is distributed under the terms of the Creative Commons Attribution 4.0 International license.

**FIG 2:**
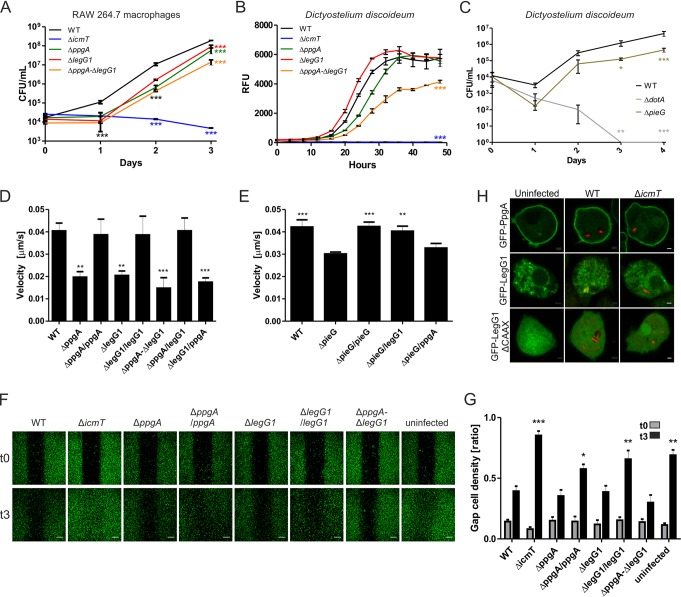
L. pneumophila RCC1 repeat effectors and their role in pathogen-host interactions. (A) Murine RAW 264.7 macrophages were infected (multiplicity of infection [MOI] 0.1) with L. pneumophila strain JR32, Δ*icmT*, Δ*ppgA*, Δ*legG1*, or Δ*ppgA*-Δ*legG1*, the cells were lysed, and intracellular replication at 37°C was assessed by CFU counting. (B) D. discoideum was infected (MOI 10) with L. pneumophila strain JR32, Δ*icmT*, Δ*ppgA*, Δ*legG1*, or Δ*ppgA*-Δ*legG1* producing GFP (pNT28), and intracellular replication at 25°C was assessed by GFP fluorescence increase. Data show means and standard deviations of results from triplicates (one-way analysis of variance [ANOVA]; ***, *P < *0.001). RFU, relative fluorescence units. (C) D. discoideum was infected (MOI 0.1) with wild-type L. pneumophila strain Paris or with the Δ*dotA* or Δ*pieG* mutant strain, and intracellular replication at 25°C was assessed by CFU counting in lysates of infected cells (3 independent experiments; two-way ANOVA; *, *P < *0.1; **, *P < *0.01; ***, *P < *0.001). (D and E) Real-time fluorescence microscopy of LCV motility in D. discoideum producing calnexin-GFP (pCaln-GFP) infected (MOI 5, 1 to 2 h) with L. pneumophila JR32, Δ*ppgA*, Δ*legG1*, or Δ*ppgA*-Δ*legG1* producing DsRed alone (pCR077) or together with M45-LegG1 (pER005) or M45-PpgA (pLS008) (D), or with the Paris wild-type strain or the Δ*pieG* mutant strain producing DsRed alone (pCR077) or together with M45-PieG (pLS033), M45-LegG1 (pER005), or M45-PpgA (pLS008) (E). LCV motility was recorded for 180 s with images taken every 10 s and quantified using ImageJ/Fiji software with the manual tracking plugin (*n* > 50/strain; 3 independent experiments; one-way ANOVA; **, *P < *0.01; ***, *P < *0.001; compared to the wild-type {D} or Δ*pieG* {E} strain). (F) D. discoideum producing GFP (pDM317) was infected (MOI 5, 1 h) with L. pneumophila JR32, Δ*ppgA*, Δ*legG1*, or Δ*ppgA*-Δ*legG1* producing DsRed alone (pCR077) or together with M45-LegG1 (pER005) or M45-PpgA (pLS008) and was seeded in a culture inset 2-well dish (Ibidi) for 2 h. After removal of the inset, cell migration was analyzed by confocal microscopy at 0 and 3 h. Bars, 200 μm. (G) Cell migration was quantified using ImageJ/Fiji software. The cell density ratio represents the average fluorescent signal intensity in the 500 μm gap divided by the average fluorescent signal intensity in 250 μm on each side of the gap center (3 independent experiments; two-way ANOVA; **, *P < *0.01; ***, *P < *0.001; all groups compared to wild-type strain at 3 h [t3]). (H) D. discoideum producing GFP-PpgA (pLS078), GFP-LegG1 (pER017), or GFP-LegG1_ΔCAAX_ (pER016) was infected (MOI 5, 1 h) with L. pneumophila JR32 or Δ*icmT* producing DsRed (pSW001), and localization of RCC1 repeat effectors was analyzed by confocal microscopy. Bars, 1 μm.

The Δ*ppgA*, Δ*legG1*, and Δ*ppgA*-Δ*legG1* mutant strains were slightly but significantly impaired for intracellular replication in murine RAW 264.7 macrophages (Fig. 2A), but not in Acanthamoeba castellanii (Fig. S3E). Upon infection of D. discoideum, the Δ*ppgA*-Δ*legG1* mutant strain was killed more efficiently at 24 h (Fig. S3F) but was impaired only for intracellular replication in a “single-round infection” assay (Fig. 2B). The Δ*pieG* mutant strain was slightly but significantly impaired for growth also in a multiple-round infection assay over several days (Fig. 2C). Upon coinfection of the Δ*ppgA*, Δ*legG1*, Δ*ppgA*-Δ*legG1*, or Δ*pieG* mutant strains with the parental strain at a 1:1 ratio in A. castellanii, the mutant strains were efficiently outcompeted by wild-type (WT) bacteria and eradicated within 6 to 9 days (Fig. S3G). Hence, PpgA—alone or in combination with LegG1—and PieG were essential for competition against wild-type L. pneumophila upon coinfection of amoebae.

L. pneumophila strains lacking *lpg1975* (LS06, Δ*lpg1975*) or *ppgA*, *legG1* and *lpg1975* (LS05, ΔΔΔ) were also outcompeted by wild-type bacteria in the amoeba competition assay (Fig. S3G), but strain Δ*lpg1975* was not impaired for intracellular replication in RAW 264.7 macrophages or A. castellanii (Fig. S3H). The competition phenotype of strain Δ*lpg1975* was not simply due to the absence of the downstream *legG1*/*lpg1976* gene, since in the mutant strain LegG1 was still produced, as established by mass spectrometry (Table S2A). Finally, *lpg1975* was not required for the Icm/Dot-dependent translocation of LegG1 or PpgA into host cells (Fig. S3I) and is not an Icm/Dot substrate itself (51). In summary, the L. pneumophila RCC1 repeat effector PpgA (in particular, together with LegG1) and PieG contribute to pathogen-host cell interactions.

10.1128/mBio.00405-20.10 TABLE S2 (A) Detection of LegG1 by mass spectrometry. (B) List of proteins identified by mass spectrometry. (C) Mass spectrometry—statistical significance. Download Table S2, XLSX file, 0.9 MB. Copyright © 2020 Swart et al. 2020 Swart et al. This content is distributed under the terms of the Creative Commons Attribution 4.0 International license.

### RCC1 repeat effectors promote LCV motility and cell migration.

LegG1 has been implicated in microtubule stability and LCV motility as well as in host cell motility (47, 53). To assess whether the L. pneumophila RCC1 repeat effector PpgA also affects pathogen vacuole motility, we infected D. discoideum producing the endoplasmic reticulum (ER)/LCV marker calnexin-GFP (calnexin-green fluorescent protein) (12) with red fluorescent L. pneumophila wild-type, Δ*ppgA*, Δ*legG1*, or Δ*ppgA*-Δ*legG1* or with the complemented strains and analyzed pathogen vacuole dynamics by live-cell fluorescence microscopy (Fig. S4A). Compared to D. discoideum infected for 1 to 2 h with wild-type L. pneumophila, LCV motility was significantly reduced by ∼50% in amoebae infected with the Δ*ppgA* mutant strain (or, as a control, the Δ*legG1* strain) (Fig. 2D). The LCV motility phenotype was complemented by providing the corresponding *ppgA* or *legG1* gene on a plasmid under the control of the P*_tac_* promoter. Interestingly, the phenotype of the Δ*ppgA* strain was reversed by plasmid-borne *legG1*, while the phenotype of the Δ*legG1* strain was not reversed by *ppgA*. This finding suggests that while both RCC1 repeat effectors promote pathogen vacuole motility, their functions are not interchangeable. Similarly, upon infection of calnexin-GFP-producing D. discoideum with L. pneumophila strain Paris or the Δ*pieG* mutant, LCV motility was reduced by approximately 25% (Fig. 2E). The LCV motility phenotype was reversed by plasmid-encoded PieG or LegG1, but not by PpgA. Taken together, the L. pneumophila RCC1 repeat effectors PpgA, PieG and LegG1 promote LCV motility, and the functions of PieG and LegG1, at least regarding LCV motility, seem to be more closely related to one another than to PpgA.

10.1128/mBio.00405-20.5 FIG S4 PpgA controls LCV motility. Download FIG S4, PDF file, 0.7 MB. Copyright © 2020 Swart et al. 2020 Swart et al. This content is distributed under the terms of the Creative Commons Attribution 4.0 International license.

To determine whether PpgA controls cell motility, we performed “gap closure” assays, where cell migration occurs to repopulate an area depleted of cells. To this end, D. discoideum producing GFP was infected with red fluorescent L. pneumophila strain JR32, Δ*ppgA*, Δ*legG1*, or Δ*ppgA*-Δ*legG1* or with the complemented strains. The infected amoebae were seeded in a culture inset 2-well dish, and after removal of the inset, cell migration was analyzed by confocal microscopy at 0 and 3 h (Fig. 2F). D. discoideum infected with the Δ*icmT* mutant as well as uninfected amoebae spread efficiently and increased the cell density in the insertion area by 70% to 90% in the course of the experiment (Fig. 2G). In contrast, D. discoideum infected with L. pneumophila strain JR32, Δ*ppgA*, Δ*legG1*, or Δ*ppgA*-Δ*legG1* barely migrated, corresponding to an increase of cell density in the insertion area of merely 25% to 35% at 3 h after the start of the assay. Upon expression of plasmid-borne *legG1* or *ppgA*, the inhibition of cell migration was reduced, in agreement with the notion that overproduction of the RCC1 repeat effectors has a positive effect on cell migration. Accordingly, the infection of amoebae with the Δ*ppgA*-Δ*legG1* mutant led to a slight (statistically not significant) “hyperinhibition” of cell migration compared to the infection with parental strain JR32. In summary, these results indicate that the RCC1 repeat effectors PpgA and LegG1 (53) promote LCV dynamics and cell motility. The effects observed for the Δ*ppgA* and Δ*legG1* mutant strains seem similar, but interestingly, in some cases they appear additive.

### PpgA is dispensable for Ran activation on LCVs and localizes to the plasma membrane.

To test whether RCC1 repeat effectors play a role for early steps of pathogen vacuole formation, D. discoideum amoebae producing the PtdIns(4)*P* probe P4C_SidC_-GFP or calnexin-GFP were infected with red fluorescent L. pneumophila strain JR32, Δ*icmT*, Δ*ppgA*, Δ*legG1*, or Δ*ppgA*-Δ*legG1*, and GFP-positive LCVs were quantified by imaging flow cytometry (IFC) (Fig. S4B). The IFC colocalization scores were identical for the parental and the RCC1 repeat effector mutant strains, indicating that PpgA and LegG1 do not affect the early steps of LCV formation.

Next, we assessed a possible role of the RCC1 repeat effectors in the activation of Ran on LCVs. To this end, we employed D. discoideum producing GFP-RanBP1, which binds only to active Ran(GTP) (47). The amoebae were infected with red fluorescent L. pneumophila strain JR32, Δ*ppgA*, Δ*legG1*, or Δ*ppgA*-Δ*legG1*, or with the complemented mutants. The ER/LCV marker calnexin was immunostained in LCV isolates of the infected cells, and the localization of GFP-RanBP1 was analyzed by confocal fluorescence microscopy (Fig. S5A). Due to a low signal-to-noise ratio in intact infected cells, the recruitment of GFP-RanBP1 was observed only in LCV isolates of infected amoebae. While around 70% of the LCVs containing JR32 or the Δ*ppgA* mutant stained positively for GFP-RanBP1, only approximately 40% of LCVs harboring the Δ*legG1* or Δ*ppgA*-Δ*legG1* mutant strain were decorated with GFP-RanBP1 (Fig. S5B). For LCVs harboring the Δ*legG1* mutant strain, the localization of GFP-RanBP1 was restored to wild-type levels by providing *legG1* on a plasmid under the control of the P*_tac_* promoter. Taken together, these findings confirm that LegG1 promotes Ran activation on LCVs (47) and indicate that PpgA is dispensable for RanBP1 accumulation on LCVs. Hence, PpgA might be targeted to another cellular compartment.

10.1128/mBio.00405-20.6 FIG S5 Ran activation on LCVs and membrane localization of RCC1 repeat effectors. Download FIG S5, PDF file, 0.5 MB. Copyright © 2020 Swart et al. 2020 Swart et al. This content is distributed under the terms of the Creative Commons Attribution 4.0 International license.

To test the hypothesis that PpgA localizes to cellular compartments other than LCVs, we used D. discoideum ectopically producing GFP-PpgA, GFP-LegG1, or GFP-LegG1_ΔCAAX_ (lacking the C-terminal prenylation motif). The amoebae were left uninfected or were infected with red fluorescent L. pneumophila JR32 or the Δ*icmT* mutant strain, and localization of RCC1 repeat effectors was analyzed by microscopy. GFP-PpgA was produced by fewer cells than GFP-LegG1 (data not shown) but localized clearly and almost exclusively to the plasma membrane in uninfected D. discoideum, as well as in amoebae infected with virulent or avirulent L. pneumophila (Fig. 2H). PpgA-dependent activation of Ran on the plasma membrane through recruitment of GFP-RanBP1 could not be observed, likely because of the low signal-to-noise ratio, which also precluded visualization of the marker on LCVs in intact infected cells (Fig. S5A). In contrast to GFP-PpgA, GFP-LegG1 showed punctate staining in the cytoplasm and localized to pathogen vacuoles harboring JR32 but not Δ*icmT* mutant bacteria, as shown previously (47). Upon removal of the C-terminal prenylation motif, GFP-LegG1_ΔCAAX_ no longer localized to LCVs in D. discoideum (Fig. 2H), in agreement with the C-terminal signature being required for the localization to specific subcellular membranes in mammalian cells (51, 52). Finally, in homogenates of infected D. discoideum, V5-tagged LegG1 and PieG accumulated on LCVs, while V5-tagged PpgA did not localize to the pathogen vacuoles (Fig. S5C). Taking the results together, while LegG1 localizes to LCVs and promotes RanBP1 accumulation (Ran activation), PpgA localizes to the eukaryotic plasma membrane rather than to LCVs and is dispensable for RanBP1 accumulation on LCVs.

### PpgA requires functional RanGAP1 to inhibit yeast growth.

For further studies on targets and the mode of action of L. pneumophila RCC1 repeat effectors, we sought to employ the yeast Saccharomyces cerevisiae, a model which has previously been successfully used to assess effects of L. pneumophila effectors on eukaryotic processes (56, 57). In particular, the use of yeast lethality suppressors has been instrumental to characterize the mode of action of some effector proteins (58). Many L. pneumophila effectors target host proteins, which are conserved among eukaryotes. The yeast small GTPase Gsp1, e.g., is 83% and 72% identical to human Ran and D. discoideum RanA, respectively.

**FIG 3:**
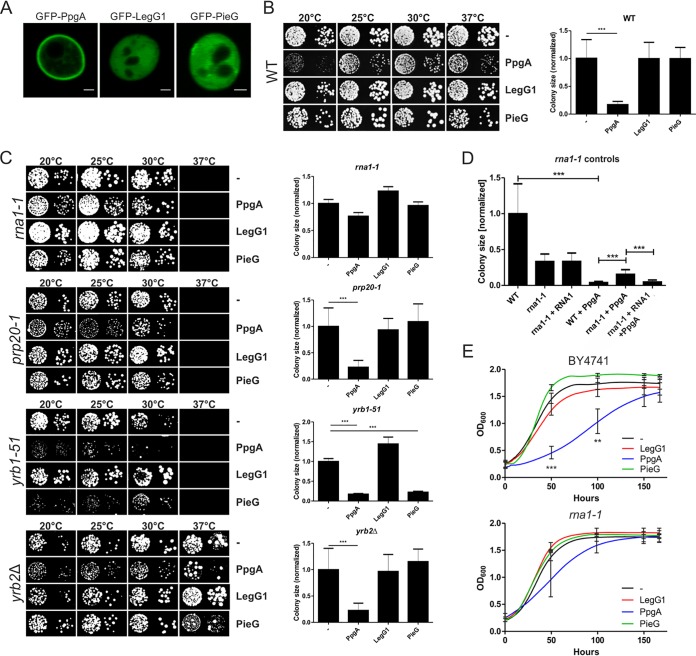
PpgA requires functional RanGAP1 to inhibit yeast growth. (A) The localization of L. pneumophila RCC1 repeat effectors was analyzed by confocal microscopy in S. cerevisiae wild-type strain BY4741 producing GFP-PpgA (pLS120), GFP-LegG1 (pLS118), or GFP-PieG (pLS113). Bars, 1 μm. (B) Growth of yeast producing RCC1 repeat proteins was tested by dot spot assays. S. cerevisiae wild-type strain BY4741 containing an empty plasmid (pYEP351gal) or producing FLAG-PpgA (pLS085), FLAG-LegG1 (pLS084), or FLAG-PieG (pLS086) was spotted in 10-fold dilutions on SG plates without leucine and grown at the indicated temperatures for 5 (30°C and 37°C), 6 (25°C), or 7 (20°C) days (left panel). Colony size (at 30°C) was measured using ImageJ/Fiji (right panel; *n* = 20; one-way ANOVA; ***, *P < *0.001). (C) S. cerevisiae mutant strains *rna1-1* (RanGAP1), *prp20-1* (RCC1), *yrb1-51* (RanBP1), and *yrb2Δ* (RanBP2) containing an empty plasmid (pYEP351gal) or a plasmid producing FLAG-PpgA (pLS085), FLAG-LegG1 (pLS084), or FLAG-PieG (pLS086) were spotted in 10-fold dilutions on SG plates without leucine and grown at the indicated temperatures for 5 (30°C), 6 (25°C and 37°C), or 7 (20°C) days (left panel). Colony size was measured using ImageJ/Fiji (right panel) (*n* > 10; one-way ANOVA; ***, *P < *0.001). (D) S. cerevisiae strain BY4741 or mutant strain *rna1-1* containing the empty plasmids pYEP351gal and pRS316, or pYEP351gal and pRS316-*RNA1* (RNA1), pRS316 and pLS085 (FLAG-PpgA), or pRS316-*RNA1* (RNA1) and pLS085 (FLAG-PpgA) was spotted in 10-fold dilutions on SG plates without leucine and uracil and grown at the indicated temperatures for 3 to 6 days. Colony size was measured using ImageJ/Fiji (one-way ANOVA; *n* > 9; ***, *P < *0.001). (E) S. cerevisiae wild-type strain BY4741 or mutant strain *rna1-1* containing the empty backbone (pYEP351gal) or producing FLAG-PpgA (pLS085), FLAG-LegG1 (pLS084), or FLAG-PieG (pLS086) was grown in SG medium without leucine for 7 days at 20°C, and OD_600_ was measured every hour. Data show means and standard deviations (for simplicity, only the standard deviations at 50 h, 100 h, 150 h, and 167 h are shown) of results from three independent experiments (two-way ANOVA; **, *P < *0.01; ***, *P < *0.001).

To validate the use of S. cerevisiae as a model to analyze RCC1 repeat effectors, we first analyzed the localization of ectopically produced GFP fusion proteins of the effectors by confocal fluorescence microscopy (Fig. 3A). GFP-PpgA predominantly localized to the plasma membrane in S. cerevisiae. In contrast, GFP-LegG1 and GFP-PieG were mostly distributed in the cytoplasm. Thus, the RCC1 repeat effectors localized to the same (PpgA) or similar (LegG1, PieG) subcellular compartments in amoebae and yeast, and accordingly, the latter might be a valid model system to functionally characterize the effectors.

Next, we tested the growth of yeast producing RCC1 repeat effectors by dot spot assays. S. cerevisiae wild-type strain BY4741 containing an empty plasmid or producing FLAG-PpgA, FLAG-LegG1, or FLAG-PieG was spotted in 10-fold dilutions on SG plates without leucine and grown at 20 to 37°C for 3 to 6 days (Fig. 3B; see also Fig. S6A). Under these conditions, production of FLAG-PpgA inhibited growth of the yeast cells, at lower temperatures in particular, while production of FLAG-LegG1 or FLAG-PieG did not affect yeast growth.

10.1128/mBio.00405-20.7 FIG S6 S. cerevisiae dot spot controls and mutant complementation. Download FIG S6, PDF file, 0.8 MB. Copyright © 2020 Swart et al. 2020 Swart et al. This content is distributed under the terms of the Creative Commons Attribution 4.0 International license.

To obtain insights into the cellular pathways mediating growth inhibition by PpgA, we employed S. cerevisiae mutant strains defective for one of the following components in the Ran GTPase cycle: *rna1-1* (RanGAP1), *prp20-1* (RCC1), *yrb1-51* (RanBP1), or *yrb2Δ* (RanBP2). FLAG-PpgA, FLAG-LegG1, or FLAG-PieG was produced in these mutant strains, and growth was assessed by dot spot assays (Fig. 3C). Interestingly, the production of FLAG-PpgA did not inhibit the growth of S. cerevisiae *rna1-1*, a temperature-sensitive mutant strain producing a nonfunctional RanGAP1 protein. Growth inhibition by FLAG-PpgA was restored upon production of wild-type RNA1 in the *rna1-1* mutant strain (Fig. 3D; see also Fig. S6B). This result indicates that growth inhibition by PpgA is mediated partially by the Ran GTPase cycle and, more specifically, requires functional RanGAP1 (RNA1). In order to further confirm the role of RNA1 for the inhibitory effect of PpgA, growth of S. cerevisiae BY4741 or *rna1-1* producing FLAG-PpgA, FLAG-LegG1, or FLAG-PieG in SG medium without leucine was monitored for 7 days at 20°C, and the optical density at 600 nm (OD_600_) was measured every hour (Fig. 3E). Under these conditions, PpgA but not LegG1 or PieG significantly slowed yeast growth in the wild-type strain, and the growth inhibition mediated by PpgA was partially alleviated in the *rna1-1* mutant strain.

Intriguingly, the production of FLAG-PieG or FLAG-PpgA also inhibited the growth of the S. cerevisiae *yrb1-51* (RanBP1) strain (Fig. 3C), suggesting that PieG and PpgA might target a RanBP1-dependent pathway. In summary, the L. pneumophila RCC1 repeat effector PpgA inhibited the growth of wild-type yeast in a RanGAP1-dependent manner, and PieG as well as PpgA inhibited the growth of a strain lacking functional RanBP1. These findings are in agreement with the notion that different components of the Ran GTPase cycle are the targets of L. pneumophila RCC1 repeat effectors.

### PpgA and LegG1 target distinct Ran GTPase cycle components.

**FIG 4:**
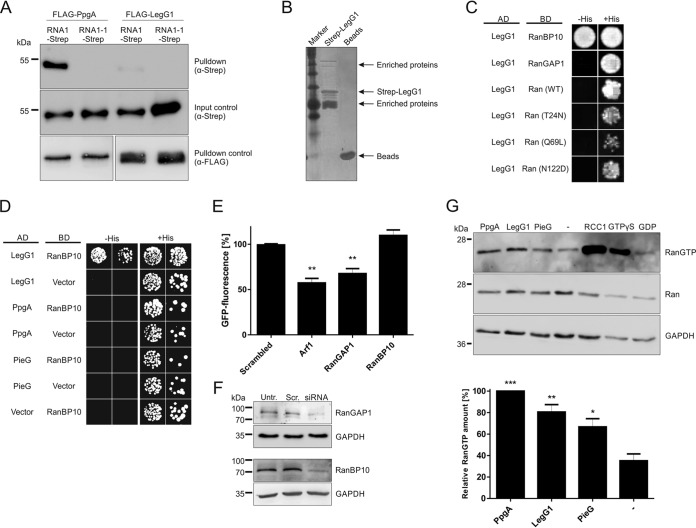
PpgA and LegG1 target distinct Ran GTPase cycle components and promote Ran activation. (A) S. cerevisiae BY4741 ectopically producing Strep-tagged RNA1 (pLS128) or RNA1-1 (pLS127) and FLAG-tagged PpgA (pLS085) or LegG1 (pLS084) was lysed, a co-IP with anti-FLAG was performed, and interactions were revealed by anti-Strep Western blotting. Yeast lysates were analyzed by Western blotting with anti-Strep (input control) and anti-FLAG (co-IP control). (B) Lysates of HEK 293T cells were incubated with Strep-LegG1_33–286_ bound to Strep-Tactin resin, and interaction partners were identified by mass spectrometry. (C) Yeast two-hybrid assays using S. cerevisiae reporter strain AH109 containing plasmids encoding the GAL4 DNA-binding domain (BD; pGBKT7) alone or fused to RanBP10 (pLS213), RanGAP1 (pM1593), Ran_WT (pM1057), Ran_T24N (pM1059), Ran_Q69L (pM1058), or Ran_N122D (pM1286) and the GAL4 activation domain (AD) fused to LegG1 (pLS211). Transformants were spotted onto SD lacking His (−His) or SD plus His (+His) and incubated at 30°C for 5 days. (D) Yeast two-hybrid assay in reporter strain AH109 containing plasmids encoding the GAL4 DNA-binding domain (BD; pGBKT7) alone or fused to RanBP10 (pLS213) and the GAL4 activation domain (AD; pGADT7) alone or fused to LegG1 (pLS211), PpgA (pM1028) or PieG (pM1027). Transformants were spotted in 10-fold serial dilutions onto SD lacking His (−His) or SD plus His (SD+) and incubated at 30°C for 5 days. (E) Human A549 cells transfected for 48 h with 10 nM siRNA oligonucleotides targeting RanGAP1, RanBP10, or Arf1 (positive control) or with AllStars siRNA (“scrambled”) were infected (MOI 10) with GFP-producing L. pneumophila JR32 (pNT28). Intracellular bacterial replication was assessed by fluorescence increase with a fluorescence plate reader after 24 h and compared to the levels seen at 1 h. Means and standard deviations of results from three independent experiments are shown (one-way ANOVA; **, *P < *0.01; all groups compared to scrambled). (F) The depletion efficiency of siRNA oligonucleotides targeting RanGAP1 or RanBP10 upon transfection of A549 epithelial cells for 48 h was assessed by Western blotting with the antibodies indicated. For each target protein four different oligonucleotides were used. Untreated cells were used as a negative control (“Untr.”), Qiagen AllStars unspecific oligonucleotides (“Scrambled,” “Scr.”) were used to control for off-target effects, and GAPDH (glyceraldehyde-3-phosphate dehydrogenase) served as the loading control. Data are representative of results from two independent experiments. (G) HEK 293T cells were transfected for 24 h with constructs producing Strep-tagged PpgA (pLS229), LegG1 (pLS226), PieG (pLS230), or RCC1 (pLS231). Nontransfected cells (-) were taken along, and lysates were treated with GTPγS (positive control) or GDP (negative control). Ran(GTP) was precipitated with RanBP1-coupled agarose beads in cell lysates, and the amount of Ran(GTP), total Ran, and GAPDH (loading control) was assessed by Western blotting (top panel). The amount of Ran(GTP) relative to total Ran was quantified using ImageQuant TL (bottom panel). Means and standard deviations of results from three independent experiments are shown for the first four bands (one-way ANOVA; *, *P < *0.05; **, *P < *0.01; ***, *P < *0.001; all groups compared to the nontransfected cells [-]).

To test the hypothesis that the RNA1-dependent growth inhibition of yeast by PpgA is due to a direct interaction between the RCC1 repeat effector and the RanGAP1 RNA1, the putative interaction partners were ectopically produced in S. cerevisiae. Specifically, we produced Strep-tagged RNA1 or the mutant protein RNA1-1 and FLAG-tagged PpgA or LegG1 in strain BY4741. The *rna1-1* allele contains two single-base-pair substitutions, resulting in two amino acid changes (S17F and A194V), which impairs enzyme activity and renders the strain temperature sensitive due to failure of RNA processing and nuclear export at the nonpermissive temperature (59). The interactions with RCC1 repeat effectors were tested by performing an anti-FLAG co-immunoprecipitation (co-IP) assay in yeast lysates, followed by anti-Strep Western blotting (Fig. 4A). Using this approach, FLAG-PpgA bound to RNA1 but not to RNA1-1, while FLAG-LegG1 did not bind to either host protein. These results indicate that PpgA but not LegG1 interacts with the RanGAP1 RNA1 and that the RNA1-1 mutations abolish the interaction. Hence, the PpgA-RNA1 interaction appears to be required for effector function.

In order to identify possible interaction partners and targets of LegG1, we incubated purified Strep-tagged LegG1_33–286_ (LegG1 lacking only the 32 N-terminal amino acids) with HEK 293T cell lysates, treated these with Strep-Tactin resin, and separated the eluate by SDS-PAGE (Fig. 4B). Under the conditions used, several proteins eluted from the beads and were identified by mass spectrometry (Table S2B and C). Among the proteins identified were Ran GTPase, RanGAP1, RanBP1, RanBP9, and RanBP10, a cytoplasmic Ran GEF implicated in the modulation of noncentrosomal microtubules (45).

To assess the interaction between L. pneumophila RCC1 repeat effectors and potential interaction partners, we employed yeast two-hybrid assays (Fig. 4C and D). To this end, we fused LegG1 to the GAL4 activation domain (AD) and RanBP10, RanGAP1, Ran (WT), or different Ran mutants to the GAL4 DNA binding domain (BD). Moreover, we fused LegG1, PpgA, or PieG to the GAL4 activation domain and RanBP10 to the GAL4 DNA binding domain. Transformants of the AH109 reporter strain were spotted in 10-fold serial dilutions onto SD lacking His (−His) or SD plus His (+His) and incubated at 30°C for 5 days. While all of the yeast strains grew in the presence of His (growth control), the strain harboring AD-LegG1 and BD-RanBP10 but not BD-RanGAP1 or BD-Ran (wild type, constitutively active, dominant negative) grew in the absence of His (Fig. 4C and D). Hence, among the putative interactions of LegG1 with Ran cycle components detected by the sensitive mass spectrometry approach, only the robust interaction with RanBP10 was validated. Under selective conditions, no growth was observed for yeast strains producing AD-PpgA or AD-PieG or strains harboring the AD or BD empty vectors. Collectively, these data confirm that LegG1 specifically binds to the cytoplasmic Ran GEF, RanBP10, but neither PpgA nor PieG does so.

### RanGAP1 is implicated in intracellular L. pneumophila replication.

Previously, we found that the depletion of Ran or RanBP1 by RNA interference reduces intracellular growth of L. pneumophila (47). To assess whether RanGAP1 and RanBP10 play a role in intracellular replication of the pathogen, the proteins were individually depleted. To this end, human A549 lung epithelial cells were treated with small interfering RNA (siRNA) oligonucleotides targeting RanGAP1, RanBP10, or Arf1 (positive control), and intracellular replication of GFP-producing L. pneumophila was monitored by fluorescence after 24 h (Fig. 4E), as previously described (37, 47, 60). Under the conditions described and in the medium used, L. pneumophila grows only intracellularly, and the GFP production (fluorescence intensity) is proportional to the number of bacteria (expressed in CFU counts). Upon depletion of RanGAP1, the fluorescence intensity of intracellular L. pneumophila was reduced approximately 2-fold, indicating that Ran inactivation plays a role in efficient intracellular growth of L. pneumophila. In contrast, the depletion of RanBP10 did not have a significant effect. The depletion of Arf1 reduced intracellular growth of L. pneumophila to a similar extent as depletion of RanGAP1. The treatment with siRNA oligonucleotides efficiently depleted RanGAP1 or RanBP10 (Fig. 4F), but had no effect on host cell viability (data not shown). Taken together, the results indicate that the depletion of RanGAP1 reduces the intracellular replication of L. pneumophila without affecting the host cell physiology, while the Ran GEF RanBP10 is dispensable or plays a redundant role for intracellular growth of the pathogen.

### Ectopic production of RCC1 repeat effectors promotes Ran activation.

Given that the L. pneumophila RCC1 repeat effectors target different components of the Ran GTPase cycle, we assessed their effect on Ran activation. To this end, we ectopically produced Strep-tagged PpgA, LegG1, PieG, or RCC1 in HEK 293T cells. After cell lysis, active Ran was precipitated with RanBP1-coupled agarose beads, and the amount of Ran(GTP) was determined by Western blotting (Fig. 4G). Upon production of the L. pneumophila RCC1 repeat proteins, the cellular amount of Ran(GTP) was increased by 2-fold to 3-fold compared to the control. As expected, overproduction of Strep-RCC1 significantly increased the amount of active Ran GTPase, as did incubation of the cleared lysate with a slowly hydrolyzable form of GTP, GTPγS, but not GDP. These results demonstrate that while the bacterial effectors target distinct components of the Ran GTPase cycle, they all promote the activation of the small GTPase, albeit less efficiently than ectopically produced RCC1.

### The Lpg1975-LegG1 fusion protein adopts PieG substrate specificity.

**FIG 5:**
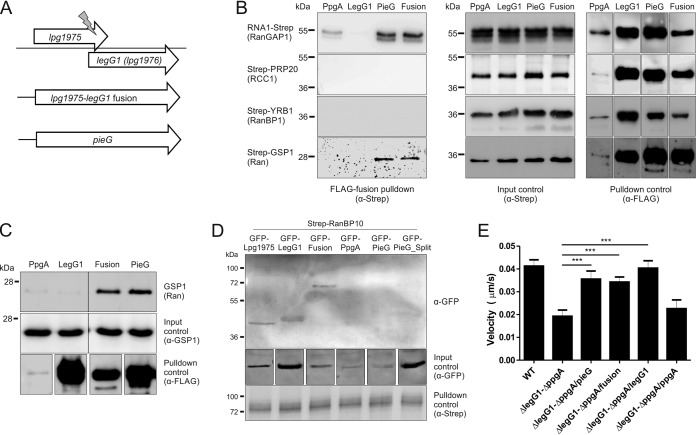
PieG and the fusion protein Lpg1975-LegG1 interact with Ran and RanGAP1. (A) A point mutation (insertion of thymidine phosphate) in L. pneumophila *lpg1975* (strain Philadelphia-1) causes a frameshift and fuses the open reading frames of *lpg1975 and legG1* (*lpg1976*), resulting in a fusion gene with high homology to *pieG* (strain Paris). (B) A co-IP screen was performed with yeast coproducing FLAG-PpgA (pLS085), FLAG-LegG1 (pLS084), FLAG-PieG (pLS086), or FLAG-Lpg1975-LegG1 fusion (pLS087) and RNA1-Strep (pLS128), Strep-PRP20 (pLS185), Strep-YRB1 (pLS186), or Strep-GSP1 (pLS173). Yeast lysates were incubated with FLAG beads, and eluates were analyzed for co-IP of Strep-proteins by anti-Strep Western blotting. Yeast lysates were analyzed by Western blotting with anti-Strep (input control) and anti-FLAG (co-IP control). (C) Lysates of yeast harboring pYEP351gal-FLAG-*ppgA* (pLS085), pYEP351gal-FLAG-*legG1* (pLS084), pYEP351gal-FLAG-*lpg1975-legG1* fusion (pLS087), or pYEP351gal-FLAG-*pieG* (pLS086) were incubated with FLAG-agarose beads, and eluates were analyzed for the co-IP of endogenous GSP1 using a specific antibody. (D) HEK 293T cells were transfected for 24 h with plasmids harboring GFP-Lpg1975 (pLS233), GFP-LegG1 (pLS227), GFP-Lpg1975-LegG1 (Fusion) (pLS234), GFP-PpgA (pLS095), GFP-PieG (pLS093), or GFP-PieG_split (pLS235) and Strep-RanBP10 (pLS242). Cell lysates were analyzed by anti-GFP Western blotting (input control), or incubated with anti-Strep antibody and A/G agarose beads, and eluates were analyzed by Western blotting for co-IP of GFP fusion proteins using anti-GFP and anti-Strep (co-IP control). (E) Real-time fluorescence microscopy of LCV motility in D. discoideum producing calnexin-GFP (pCaln-GFP) (green) infected (MOI 5, 1 to 2 h) with L. pneumophila JR32 (WT) or with the Δ*legG1*-Δ*ppgA* mutant producing DsRed alone (pCR077) or together with M45-tagged PieG (pLS033), Lpg1975-LegG1 fusion protein (pLS026), LegG1 (pER005), or PpgA (pLS008). LCV motility was recorded for 180 s with images taken every 10 s and quantified using ImageJ/Fiji software with the manual tracking plugin (*n* > 50/strain; 3 independent experiments; one-way ANOVA; ***, *P < *0.001; all groups compared to Δ*legG1*-Δ*ppgA*).

An interesting pattern seen among all but one of the sequenced L. pneumophila genomes was that if two RCC1 paralogs were present in a genome, one of them was split (Fig. 1). Thus, the single RCC1 repeat gene in strain Paris, *pieG*, was split in strain Philadelphia-1 into *legG1* and the upstream gene, *lpg1975*. A single point mutation in the gene corresponding to *pieG* (deletion of a thymidine phosphate) caused a frameshift and resulted in the two open reading frames, *lpg1975* and *legG1* (Fig. 5A). This pattern is conserved and was observed for at least 31 L. pneumophila strains (Fig. 1). Reverting the frameshift mutation, we created the *lpg1975*-*legG1* fusion by insertion of a thymidine phosphate. The corresponding Lpg1975-LegG1 fusion protein (strain Philadelphia-1) is approximately 90% identical to PieG (strain Paris) (Fig. S7). In order to characterize potential functional consequences of the point mutation leading to split PieG, we assessed the host cell localization and targets of the L. pneumophila RCC1 repeat effectors.

10.1128/mBio.00405-20.8 FIG S7 Alignment of L. pneumophila RCC1 repeat genes. Download FIG S7, PDF file, 1.4 MB. Copyright © 2020 Swart et al. 2020 Swart et al. This content is distributed under the terms of the Creative Commons Attribution 4.0 International license.

Similarly to LegG1 from strain Philadelphia-1, PieG from strain Paris localizes to LCVs (Fig. S5C). In order to identify potential interaction partners and targets of the RCC1 repeat effectors, a co-IP screen was performed with yeast producing FLAG-tagged PpgA, LegG1, PieG, or Lpg1975-LegG1 (Fusion) and RNA1-Strep (RanGAP1), Strep-PRP20 (RCC1), Strep-YRB1 (RanBP1), or Strep-GSP1 (Ran). Yeast lysates were incubated with anti-FLAG beads, and eluates were analyzed for co-IP of Strep-tagged host proteins by anti-Strep Western blotting (Fig. 5B). Strikingly, FLAG-PieG as well as the FLAG-Lpg1975-LegG1 fusion protein, but not FLAG-LegG1, bound both RNA1-Strep (RanGAP1) and Strep-GSP1 (Ran). As observed before, FLAG-PpgA interacted with RNA1-Strep (RanGAP1). Under these conditions, LegG1 did not bind to any of the Ran GTPase cycle components tested. These results indicate that the Lpg1975-LegG1 fusion protein created upon insertion of a single nucleotide targets the same Ran GTPase cycle components as PieG.

To confirm these results with endogenous GSP1 (Ran), we produced FLAG-tagged PpgA, LegG1, Lpg1975-LegG1 (Fusion), or PieG in the S. cerevisiae wild-type strain and incubated lysates with FLAG-agarose beads. Eluates from the beads were analyzed for the co-IP of endogenous GSP1 using an anti-GSP1 antibody (Fig. 5C). Under these conditions, FLAG-PieG as well as the FLAG-Lpg1975-LegG1 fusion protein precipitated GSP1 (Ran), while LegG1 did not.

In order to assess the RCC1 repeat effector targets in mammalian cells, we performed co-IP experiments in HEK 293T cells (Fig. 5D). To this end, the cells were transfected with a plasmid producing Strep-RanBP10 and plasmids producing GFP-Lpg1975, GFP-LegG1, GFP-Lpg1975-LegG1 (Fusion), GFP-PpgA, GFP-PieG, or GFP-PieG_split (corresponding to LegG1). Cell lysates were incubated with anti-Strep antibody and protein A/G agarose beads, and eluates were analyzed for co-IP of GFP using an anti-GFP antibody. Under these conditions, RanBP10 was bound by LegG1, validating the binding studies performed in yeast (Fig. 4). Moreover, RanBP10 was weakly bound by Lpg1975 as well as by the Lpg1975-LegG1 fusion protein, but not at all by PieG or the C-terminal part of PieG (PieG_split), which corresponds to but is not identical with LegG1.

Finally, to assess whether the Lpg1975-LegG1 fusion protein can functionally replace PieG, we sought to complement the LCV motility phenotype of the Δ*legG1*-*ΔppgA* double mutant strain (Fig. 5E). D. discoideum producing the ER/LCV marker calnexin-GFP was infected with the red fluorescent L. pneumophila wild-type strain or with the Δ*legG1*-Δ*ppgA* mutant producing M45-tagged PieG, Lpg1975-LegG1 fusion protein, LegG1, or PpgA, and LCV motility was recorded by fluorescence microscopy. The LCV motility defect of the Δ*legG1*-*ΔppgA* double mutant strain was restored to similar extents by Lpg1975-LegG1, PieG, and LegG1, indicating that the fusion protein is functional.

**FIG 6:**
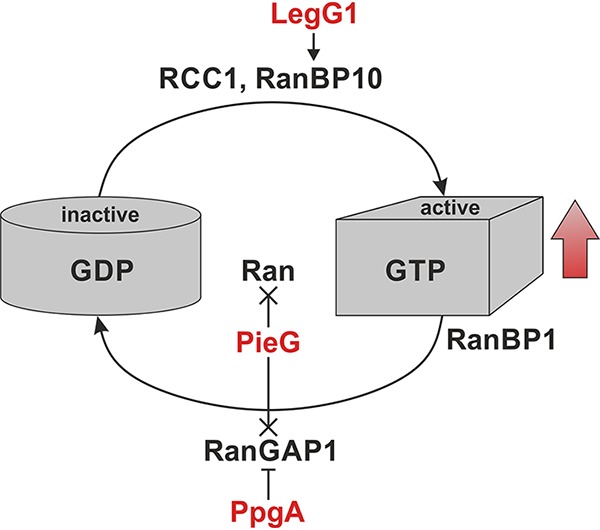
Ran GTPase cycle targets of L. pneumophila RCC1 repeat effectors. The different Ran GTPase cycle targets of the L. pneumophila RCC1 repeat effectors LegG1, PpgA and PieG (red) are indicated. Divergent evolution of L. pneumophila RCC1 repeat effectors defines the range of target components of the Ran GTPase cycle. Regardless of the distinct target, the L. pneumophila RCC1 repeat effectors promote the activation of Ran. Putative activation (>), inhibition (|), or binding (×) of Ran GTPase cycle components by L. pneumophila RCC1 repeat effectors is indicated with different arrow endings. For details see text.

In summary, the results of this study indicate that the divergent evolution of L. pneumophila RCC1 repeat effectors defines the range of Ran GTPase cycle targets. Accordingly, PpgA targets RanGAP1, LegG1 binds RanBP10, and PieG as well as the Lpg1975-LegG1 fusion interact with Ran and RanGAP1 (Fig. 6; see also Table 1). The L. pneumophila RCC1 repeat effectors promote the activation of Ran GTPase, and thus, our working model stipulates that LegG1 activates the GEF RanBP10, PpgA inhibits RanGAP1, and PieG stabilizes Ran(GTP) and/or inhibits RanGAP1.

**TABLE 1:** Phenotypes of *Legionella* RCC1 repeat effectors

Phenotype	Characteristic		
	PpgA	LegG1	PieG
LCV motility	Increased	Increased	Increased
Cellular localization	Plasma membrane	LCV membrane	LCV membrane
Cell migration	Increased	Increased	Not tested
Yeast growth (wild-type)	Reduced	Not affected	Not affected
Ran cycle target(s)	RanGAP1	RanBP10	Ran and RanGAP1

## DISCUSSION

The presence of many “eukaryote-like” genes in the L. pneumophila genome (i.e., genes whose closest homologue is found in eukaryotes) reflects an intimate relationship and exchange of genetic material between the facultative intracellular pathogen and its protozoan hosts (48–50). In this study, we investigated the evolution and functions of *Legionella* effectors containing the eukaryotic RCC1 repeat, which is implicated in the activation of the small GTPase Ran. Bioinformatics revealed that RCC1 repeat genes are present in many *Legionella* species (24 of 58 analyzed). Only a few, if any, of these RCC1 genes appeared to be orthologues of *lpp1959* (*pieG*). In any case, none of these RCC1 genes was shorter than *lpp1959*, ruling out the presence of a split *pieG* gene in *Legionella* species other than L. pneumophila.

The RCC1 repeat genes are conserved in all L. pneumophila strains analyzed (Fig. 1). The strains harboring one RCC1 repeat effector gene (e.g., *pieG* in strain Paris or Lens) do not form a single cluster in the phylogenetic tree. In contrast, strains harboring two RCC1 repeat genes (a split *pieG*: *legG1*, and *ppgA*) form a distinct cluster and thus share a common ancestor. In case of split *pieG*, only one gene (*legG1*) contains a predicted RCC1 repeat. A few strains in the Lens-Paris cluster form a subcluster and contain *pieG* and in addition two or three copies of *ppgA*. This subcluster likely acquired the *ppgA* gene(s) after *pieG*. On the basis of the overall phylogeny, an ancestral L. pneumophila strain harboring *pieG* might have acquired the *ppgA* gene(s). However, we cannot exclude the possibility that the RCC1 repeat gene distribution in L. pneumophila is the result of an ancestral strain harboring both *pieG* and *ppgA*, where duplication of *ppgA* took place in some strains, loss of *ppgA* in others, and retention and splitting of *pieG* in the majority.

Interestingly, when two RCC1 repeat genes are present in a L. pneumophila genome, one of them is split. On the basis of the available data, we cannot deduce whether in the course of genome evolution the split of *pieG* (yielding *legG1* and *lpg1975*) or the acquisition of *ppgA* happened first. While *pieG* and *ppgA* occur together in strain HL06041035, in this case one *ppgA* copy also suffered a major mutation (internal deletion). Hence, there seems to be considerable evolutionary pressure on the RCC1 repeat genes, originating either from the challenging host cell interactions (see below) or from the requirements to produce a eukaryotic protein in a prokaryote. In any case, L. pneumophila as well as Escherichia coli and Yersinia enterocolitica efficiently produce the “trimmed” version of PieG, LegG1. Moreover, Y. enterocolitica was found to produce active LegG1, which upon delivery into eukaryotic cells promoted microtubule stabilization (47). Given that LegG1 promotes microtubule stabilization upon this “microbial microinjection” by Y. enterocolitica and that the L. pneumophila Δ*legG1* mutant strain has strong and pleiotropic phenotypes, the RCC1 repeat gene is likely not on the path to pseudogenization.

The deletion of a single nucleotide in the gene of strain Philadelphia-1 corresponding to *pieG* of strain Paris leads to a frameshift and, consequently, to two open reading frames in the genome of Philadelphia-1: *lpg1975* and *legG1* (Fig. 5A). The consequences of this point mutation are remarkable on the protein level. While PieG (53 kDa) binds Ran as well as RanGAP1, LegG1 (31 kDa) specifically binds the cytoplasmic Ran GEF, RanBP10 (Fig. 4 and 5). Strikingly, this substrate switch can be experimentally reversed by introducing an additional nucleotide in the *lpg1975*-*legG1* sequence, thus restoring the production of the Lpg1975-LegG1 fusion protein with the binding characteristics of PieG (Fig. 5).

Ran is a pleiotropic small GTPase implicated in diverse cellular processes such as nucleocytoplasmic transport as well as mitotic and nonmitotic microtubule dynamics (39, 40). The depletion by RNA interference of Ran or its effector RanBP1 (binding only to activated Ran) inhibits the intracellular replication of L. pneumophila, and both Ran and RanBP1 localize to LCVs (47). The prenylated Icm/Dot substrate LegG1 promotes local Ran activation on LCVs, microtubule stabilization, and LCV motility (47). The sustained LCV dynamics along microtubules likely uphold the interactions of the pathogen vacuole with cellular vesicles throughout the bacterial infection. Moreover, LegG1 induces mitochondrial fragmentation during infection and thus also seems to affect microtubule dynamics at a distance from the pathogen compartment (54). Finally, LegG1 stimulates the chemotactic migration of D. discoideum amoebae, macrophages, and neutrophils (53). Yet it is not clear whether this effect has direct implications for L. pneumophila virulence or whether it is an indirect consequence of microtubule stabilization.

Given the pleiotropic functions of Ran GTPase, its unrestricted activation is likely detrimental to host cells. In agreement with this notion, ectopic production of PpgA in yeast inhibited the growth and was potentially toxic for the cells (Fig. 3B; see also Fig. S6A in the supplemental material). In order to avoid a potentially disruptive activation of Ran, its subversion by a pathogen must be tightly controlled regarding the specificity, localization, secretion hierarchy, and half-live of the effector(s). Our results revealed that PpgA interacted with RanGAP1, LegG1 bound the cytosolic GEF RanBP10, and PieG bound Ran GTPase as well as RanGAP1 (Fig. 4 and 5). Thus, effector substrate expansion due to the presence of two or more RCC1 repeat effectors in a given L. pneumophila strain might fine-tune Ran activation during *Legionella* infection, and strains harboring distinct combinations of RCC1 repeat effectors might affect Ran activation differently. Targeting of Ran modulators allows the effectors to exploit the inherent specificity of these modulators, and thus, several layers and levels of control can be achieved. Moreover, the injection timing and hierarchy as well as the host cell half-life of L. pneumophila effectors might affect Ran activation; however, these intricate and complex issues have not been addressed thus far.

Furthermore, we provide evidence for distinct manifestations of spatial control, i.e., different subcellular localizations of L. pneumophila RCC1 repeat effectors. LegG1 and PieG localize to the LCV membrane, and this localization is dependent on the C-terminal CAAX prenylation motif (Fig. 2H; see also Fig. S5C) (47, 52). In contrast, PpgA exclusively localizes to the plasma membrane in D. discoideum amoebae as well as in the yeast model (Fig. 2H and 3A). The specific localization of PpgA and its binding to RanGAP1 suggest a function of Ran at the plasma membrane which to our knowledge has not been described before. Alternatively, RanGAP1 might have Ran-independent functions at the plasma membrane. The subcellular localization of PpgA at the plasma membrane is congruent with a role in cell migration and chemotaxis (Fig. 2G). An elaborate form of spatiotemporal control of Ran activation by different L. pneumophila RCC1 repeat effectors might also explain the apparently counterintuitive finding that depletion of Ran as well as depletion of its inhibitor, RanGAP1, reduced intracellular growth of L. pneumophila (Fig. 4E) (47). Again, these findings are also in agreement with Ran-independent functions of RanGAP1. Taken together, in addition to substrate expansion, different subcellular localizations of L. pneumophila RCC1 repeat effectors might contribute to control Ran activation in infected host cells. A sophisticated and fine-tuned form of spatiotemporal control of Ran activation might indeed be the evolutionary driving force behind the acquisition of the second RCC1 repeat effector, PpgA, in the L. pneumophila Philadelphia-C7O cluster (Fig. 1).

At this point, we cannot rule out the possibility that PpgA also functions at other sites in the cell. The motility of LCVs harboring a Δ*ppgA* mutant strain was reduced similarly to that of LCVs harboring Δ*legG1* (Fig. 2D; see also Fig. S4A). However, PpgA appeared to be dispensable for Ran activation directly on LCVs, since Ran was still activated on the pathogen vacuoles harboring Δ*ppgA* (as judged by accumulation of GFP-RanBP1) (Fig. S5A and B). The different subcellular localizations (and possibly functions) of PpgA and LegG1 are also reflected in the fact that plasmid-borne *legG1* restored the LCV motility phenotype of the Δ*ppgA* mutant strain whereas *ppgA* had no effect on the corresponding Δ*legG1* phenotype (Fig. 2D; see also Fig. S4A). Notably, these findings are also in agreement with the presence of a Ran activation pathway where PpgA precedes LegG1. In addition to its role in pathogen vacuole motility and microtubule stabilization, LegG1 (alias MitF) has been implicated in microtubule-dependent organelle dynamics. LegG1 promotes the fission of the mitochondrial network through the accumulation of a mitochondrial large GTPase, DNM1L. Mitochondrial fission halts respiration and shifts the host cell metabolism toward glycolysis in a process called the “Warburg effect.”

The L. pneumophila RCC1 repeat effectors LegG1, PpgA, and PieG play an important role in pathogen-host interactions (Fig. 2; see also Fig. S3) (47) and, upon ectopic production, function as bacterial Ran activators [i.e., cause the cytoplasmic levels of Ran(GTP) to increase], albeit less efficiently than RCC1 (Fig. 4G) (47). Perhaps the RCC1 repeat effectors are less efficiently produced in eukaryotic cells, which might at least partially account for their rather inefficient Ran activation, or perhaps they are less efficient than RCC1, because they indirectly activate Ran by targeting Ran-modifying proteins. Plausible mode of actions for the different RCC1 repeat effectors to increase cellular levels of Ran(GTP) are that LegG1 activates the GEF RanBP10, PpgA inhibits the GAP RanGAP1, and PieG and the Lpg1975-LegG1 fusion protein stabilize Ran(GTP) by binding to the active GTPase and/or inhibit RanGAP1 (Fig. 6; see also Table 1).

The further investigation of the mode of action of RCC1 repeat effectors requires their purification and biochemical analysis. However, our attempts to purify PpgA or PieG from E. coli or from L. pneumophila overproducing the effectors have thus far remained unsuccessful. Production of various recombinant fusion proteins, including His, GST, GFP, and MBP fusions, at different temperatures resulted in large amounts of aggregated insoluble protein. Efforts to improve protein folding, e.g., coproduction of bacterial chaperones, failed to increase the yield of soluble protein. In summary, on the basis of bioinformatic, genetic, and cell biological insights, this study shed light on the divergent evolution of L. pneumophila RCC1 repeat proteins, leading to an expansion of their substrate range and thus possibly allowing fine-tuning of Ran activation. This work paves the way for further mechanistic analysis of L. pneumophila RCC1 repeat effectors.

## MATERIALS AND METHODS

See Text S1 in the supplemental material for additional detailed descriptions of the materials and methods used in the study.

10.1128/mBio.00405-20.1 TEXT S1 Supplemental methods and references. Download Text S1, PDF file, 0.2 MB. Copyright © 2020 Swart et al. 2020 Swart et al. This content is distributed under the terms of the Creative Commons Attribution 4.0 International license.

### Bioinformatics analysis.

The Pfam tool (61) was used for the identification of RCC1 proteins in L. pneumophila genomes. A phylogenetic tree of the 59 fully sequenced L. pneumophila genomes examined in this study was constructed using Parsnp, a fast core-genome multialignment tool, with default parameters, removing recombinant regions (62). The orthologous relationships among the proteins of all the selected strains were obtained by running OrthoMCL (63) with an inflation index of 2.5.

### Strains and plasmids.

The L. pneumophila and S. cerevisiae strains used in this study are listed in Table S1A in the supplemental material. Cultivation and transformations were performed according to established protocols. Construction of L. pneumophila mutant strains was performed as previously described (64). Plasmids and oligonucleotides used in this study are listed in Table S1A and B, respectively. All recombinant DNA techniques were employed according to established procedures using E. coli TOP10 cells.

### Host cell infection.

L. pneumophila strains (Table S1A) were grown on charcoal-yeast extract (CYE) agar plates. For infection, liquid cultures in ACES [*N*-(2-acetamido)-2-aminoethanesulfonic acid] yeast extract (AYE) medium were inoculated at an OD_600_ of 0.1 and grown at 37°C to the early stationary phase. Chloramphenicol (Cam; 5 μg/ml) and isopropyl-β-d-thiogalactopyranoside (IPTG; 1 mM) were added as required. Cultures were diluted to the desired density (multiplicity of infection [MOI] 0.1 to 50), infections of phagocytes were synchronized by centrifugation (450 × *g*, 10 min at room temperature [RT]), and infected cells were incubated at the indicated temperature for the indicated time. Intracellular replication and competition assays were performed as described in Text S1.

### (Real-time) fluorescence microscopy.

Microscopy was performed with a Leica SP8 inverse laser scanning confocal microscope using D. discoideum amoebae or S. cerevisiae producing GFP fusion proteins. Live-cell experiments were performed in imaging dishes (Ibidi), or cells were fixed on poly-l-lysine-coated coverslips and stained with primary and secondary antibodies as indicated. Intact LCVs were purified by the two-step immunoaffinity procedure as described previously (36, 37).

### Imaging flow cytometry.

D. discoideum cells producing GFP fusion proteins were infected with DsRed-producing L. pneumophila strains and analyzed at the indicated time points by IFC (ImageStream X Mk II; Amnis) as previously described (65).

### Yeast methods.

For two-hybrid and spot assays, yeast wild-type or mutant strains (Table S1A) were grown to the logarithmic phase, and 10-fold dilutions were spotted on SD or SG plates as indicated. Yeast growth was documented by camera, and colony size was quantified by ImageJ.

### Co-immunoprecipitation.

FLAG-tagged and Strep-tagged proteins were coproduced in yeast and affinity purified using anti-FLAG M2 affinity gel (Sigma) according to the manufacturer’s protocol. Samples were analyzed for the presence of endogenous or Strep-tagged proteins by Western blotting as described in detail in Text S1. Strep-tagged LegG1_33–286_, purified from E. coli, was preincubated with HEK 293T cell lysate and subsequently loaded on Strep-Tactin Sepharose (IBA). LegG1 and interaction partners were eluted with desthiobiotin, subjected to trichloroacetic acid (TCA) precipitation, separated by SDS-PAGE, and analyzed by mass spectrometry. To confirm the interaction between LegG1 and RanBP10 observed by yeast two-hybrid assay, HEK 293T cells were cotransfected using Lipofectamine 3000 reagent (Invitrogen) with plasmids, producing GFP-tagged Lpg1975, LegG1, Lpg1975-LegG1 (fusion), PpgA, PieG, or PieG_190–475_ and Strep-tagged RanBP10. The co-IP was performed using an antibody against Strep tag and A/G agarose beads.

### Mass spectrometry analysis.

For protein identification, tryptic peptides were separated and analyzed by nano-high-performance liquid chromatography (nano-HPLC)/tandem mass spectrometry (MS/MS), and data evaluation was performed using MaxQuant software (66) (v.1.5.3.30) or Perseus software (67) (v. 1.5.2.6).

### Ran activation assay.

The cellular amount of Ran(GTP) was analyzed using a Ran activation assay kit (Cell Biolabs). HEK 293T cells were transfected for 24 h with pEGFP derivatives using Lipofectamine 3000 (Invitrogen). Cells were lysed, and Ran(GTP) was precipitated by the use of RanBP1 PBD agarose beads. Proteins were eluted from beads and analyzed by Western blotting as described in detail in Text S1.

### RNA interference and determination of protein depletion efficiency.

RNA interference experiments were performed as described previously (47, 60), and the siRNA oligonucleotides used are listed in Table S1C.

### Data availability.

All data are available in the main text or in Text S1 (provided as source data files).
