# Supplementary material for: Divergent Evolution of Legionella RCC1 Repeat Effectors Defines the Range of Ran GTPase Cycle Targets
Source: mBio. 2020 Mar 24;11(2):e00405-20. doi: 10.1128/mBio.00405-20 (PMC7157520; doi:10.1128/mBio.00405-20)
Supplement: FIG S1 [file mBio.00405-20-sf001.pdf]

Figure S1

A

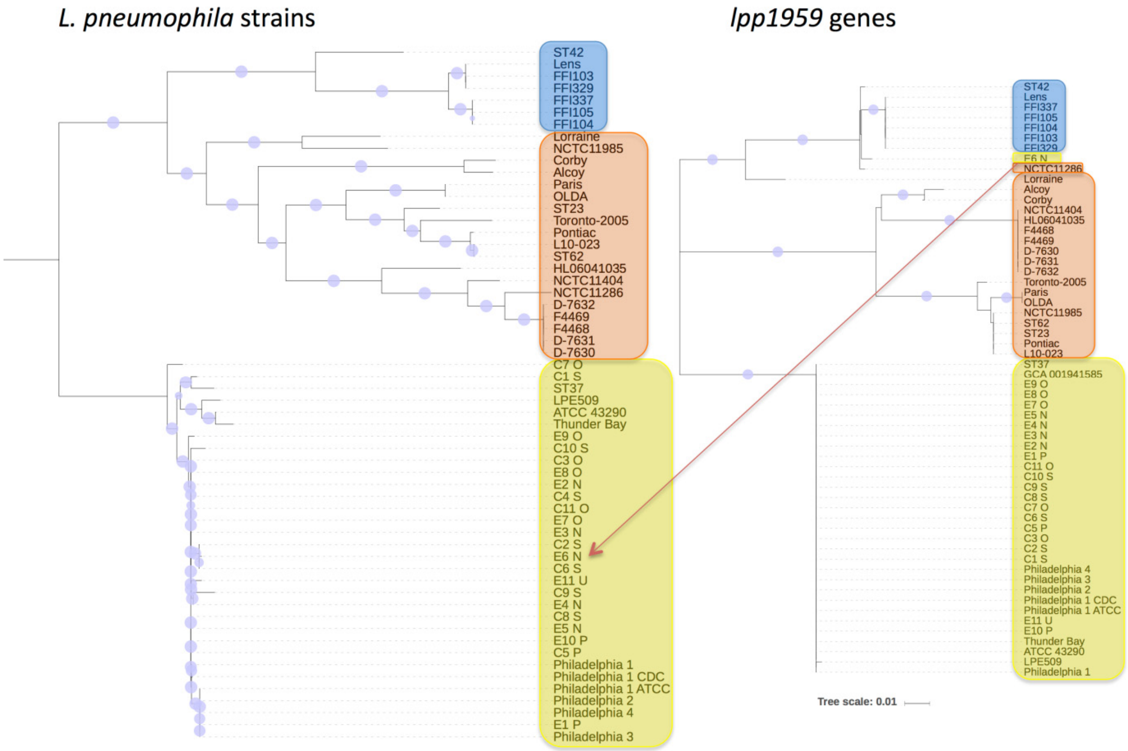

L. pneumophila strains
lpp1959 genes
ST42
Lens
FFI103
FFI329
FFI337
FFI105
FFI104
Lorraine
NCTC11985
Corby
Alcoy
Paris
OLDA
ST23
Toronto-2005
Pontiac
L10-023
ST62
HL06041035
NCTC11404
NCTC11286
D-7632
F4469
F4468
D-7631
D-7630
C7 O
C1 S
ST37
LPE509
ATCC 43290
Thunder Bay
E9 O
C10 S
C3 O
E8 O
E2 N
C4 S
C11 O
E7 O
E3 N
C2 S
E6 N
C6 S
E11 U
C9 S
E4 N
C8 S
E5 N
E10 P
C5 P
Philadelphia 1
Philadelphia 1 CDC
Philadelphia 1 ATCC
Philadelphia 2
Philadelphia 4
E1 P
Philadelphia 3
ST42
Lens
FFI337
FFI105
FFI104
FFI103
FFI329
E6 N
NCTC11286
Lorraine
Alcoy
Corby
NCTC11404
HL06041035
F4468
F4469
D-7630
D-7631
D-7632
Toronto-2005
Paris
OLDA
NCTC11985
ST62
ST23
Pontiac
L10-023
ST37
GCA 001941585
E9 O
E8 O
E7 O
E5 N
E4 N
E3 N
E2 N
E1 P
C11 O
C10 S
C9 S
C8 S
C7 O
C6 S
C5 P
C3 O
C2 S
C1 S
Philadelphia 4
Philadelphia 3
Philadelphia 2
Philadelphia 1 CDC
Philadelphia 1 ATCC
E11 U
E10 P
Thunder Bay
ATCC 43290
LPE509
Philadelphia 1
Tree scale: 0.01

B

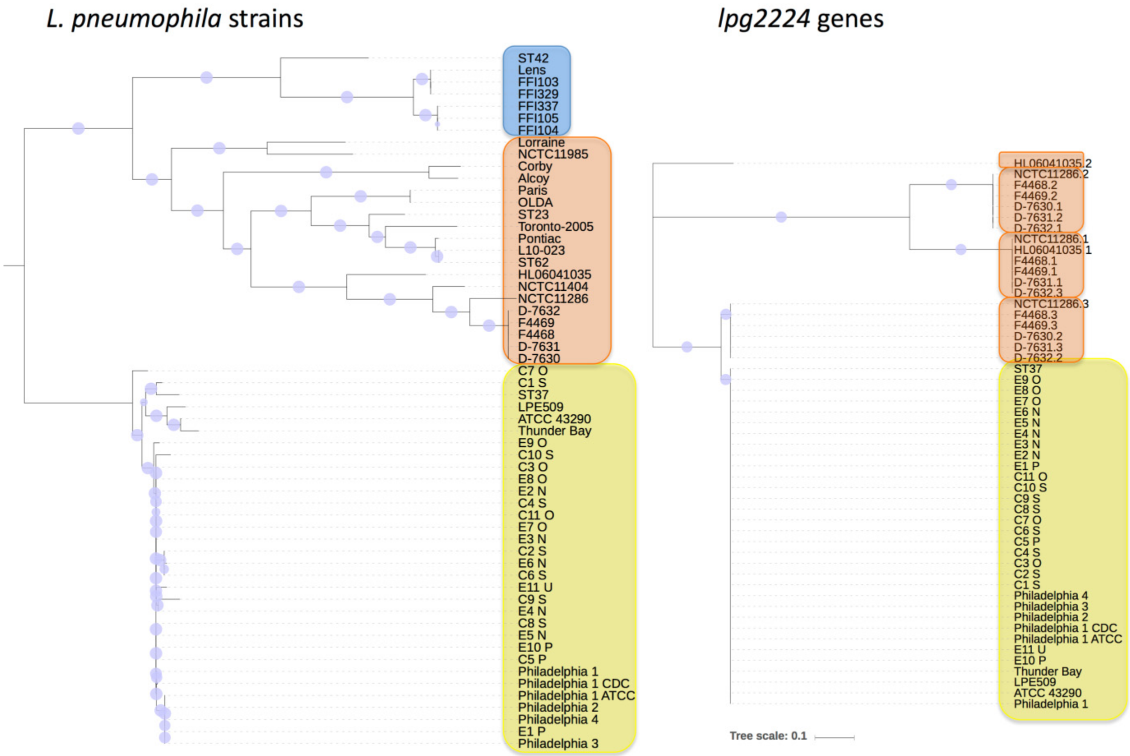

L. pneumophila strains
lpg2224 genes
ST42
Lens
FFI103
FFI329
FFI337
FFI105
FFI104
Lorraine
NCTC11985
Corby
Alcoy
Paris
OLDA
ST23
Toronto-2005
Pontiac
L10-023
ST62
HL06041035
NCTC11404
NCTC11286
D-7632
F4469
F4468
D-7631
D-7630
C7 O
C1 S
ST37
LPE509
ATCC 43290
Thunder Bay
E9 O
C10 S
C3 O
E8 O
E2 N
C4 S
C11 O
E7 O
E3 N
C2 S
E6 N
C6 S
E11 U
C9 S
E4 N
C8 S
E5 N
E10 P
C5 P
Philadelphia 1
Philadelphia 1 CDC
Philadelphia 1 ATCC
Philadelphia 2
Philadelphia 4
E1 P
Philadelphia 3
HL06041035.2
NCTC11286.2
F4468.2
F4469.2
D-7630.1
D-7631.2
D-7632.1
NCTC11286.1
HL06041035.1
F4468.1
F4469.1
D-7631.1
D-7632.3
NCTC11286.3
F4468.3
F4469.3
D-7630.2
D-7631.3
D-7632.2
ST37
E9 O
E8 O
E7 O
E6 N
E5 N
E4 N
E3 N
E2 N
E1 P
C11 O
C10 S
C9 S
C8 S
C7 O
C6 S
C5 P
C4 S
C3 O
C2 S
C1 S
Philadelphia 4
Philadelphia 3
Philadelphia 2
Philadelphia 1 CDC
Philadelphia 1 ATCC
E11 U
E10 P
Thunder Bay
LPE509
ATCC 43290
Philadelphia 1
Tree scale: 0.1

Fig. S1 (overleaf). Comparison of phylogenetic trees of *L. pneumophila* strains and RCC1 repeat effector genes. The same or similar clusters appear in the phylogenetic trees of *L. pneumophila* strains and the (**A**) *lpp1959* (*pieG*) or (**B**) *lpg2224* (*ppgA*) genes. Circles at nodes represent bootstrap support, and size of circle is proportional to bootstrap value. Only bootstrap values above 80% are shown. Scale bar represents the estimated number of substitutions per site.
