# Supplementary material for: Divergent Evolution of Legionella RCC1 Repeat Effectors Defines the Range of Ran GTPase Cycle Targets
Source: mBio. 2020 Mar 24;11(2):e00405-20. doi: 10.1128/mBio.00405-20 (PMC7157520; doi:10.1128/mBio.00405-20)
Supplement: FIG S2 [file mBio.00405-20-sf002.pdf]

Figure S2

A

>PpgA
MKEPRELNRQPNPEHMAQLIKDTPKDVWFHLAKYLPDHELLNLMQVDKRLNRFFKEGTPPKEIIKKIEHAPLKVVSSGVNTFILTQYHGKPLLLACGQNKDGQLGCGDTKIR
TALTPVNIPESFASIESVQISLFHAVLWGRDKDNKPILATCGLNDKGQLGCGDYKSRALFTQVKLAEDITSIESVEINIFHSVILGHDKHNRPVLASCGSNHLGKLGCGDIKDKTTF
TRMKLPTEIVSIDTLQMNWHTIVSGRDKNNKPVLAACGWNDRGQLGCGDKKERALLTPVKLPENIVSIQSAQIGTSRTTVLGRDKDNKPILTYCGWNERNQMGWSNTEDK
TALTPAKLPENIVSIELMQISTSRTVILGRDKNNKPILAACGLNNHGQLGCGDIKTRTTLYPIKFPKDIVSIESVQISDNHTVILGRDKHKNPIIAACGLNNYGQLGCGDTKNRMLL
TPIKLPKEIVCIDSVTVKYSSLFVSGRDKNHRPILAACGDNRYGQLGVPSKKLLTELTIVPTPILTLPATPKREASSRKEATEKALVFATHGSREQKPRKSNRILTAIIASFGRFFTGAH
PHTPDSDKESPSPKRSA

>LegG1
LHLELHQINGNDPLGTIIGERNYQLPIPLPYTMKTVKQIHSGIWHTVIRGYDQYNRPQIATFGKNYDGQLGTGDCENRDTPTPIKLPPEMLTFHAIQTGFFHTIATGLDKNGFPI
VASCGYNSDGQLGCGDKDNKNTLIPIKLPENMKSVEMVATGVKHTVIVGKDINNKPVVATCGHNGYGQLGTGDEENRLCLTPIGIPEDITSVDYVGAGAYHTVICGRNIKNQP
IITLCGCNSDGELGFAPQKIAKKSSLMTNPAMFFPPRQDKSAINNLMKISKFSPCNLL

>Lpg1975
VGYNETIYHKPFIKINMPDVLGKPDATQIPDDVWLDILINYLQPQDIVNLSEINNRLSRLFKTQHTQSEQLNQKKTLGILKVFAGYSSTFLFARLNGKQRLFKTRNWSAADSDNI
NPILMPISLPENISVIESIQSTYKHTVVVGRDKHDQIIVATNKINQDYAKQETSHNQLIPLPLRDINISLVSAGNEEDYILNPIFSFPATQQGNCILNCIK

>PieG
MPDVSGKPDANQIPDDVWLDILINYLQPQDAVNLSEVNNRLNRLFATQHTQSEQLHRKKTLGILKVFTGYSSTFLFIRFNGKQRLFRAMDSNSITPLLMPISLPENVSYIESIQST
YKHTVVVGRDNRDQIIVATNKIHKDYSRQETSHNQLIPLPLRNNNISLVSVGNNEESYIFNPIFLLSRNAAGQLSLQLHKINSKAPLETTIEDRDCQLPIPLPDTFKTVTKISSGVWH
TVIAGYDQHNRPQIATFGKNCDGQLGTGDWENRDTPTQAKLPPEMHTLHTIQTRFFHTIATGLDKNGYPIVASCGYNSDGQLGCGDKDNKNALIPIKLPENMKSVEMVATGV
KHTVIVGKDINNKPMVATCGHNGYGQLGTGDEENRLYLTLIGIPEDITTVDYIEAGTYHTVICGRNMKNQPTLALCGCNSDGELGLAPQKITQKSSLMTNPALFFPPRQDRNAIN
NMMKISTFSPCNLL

B

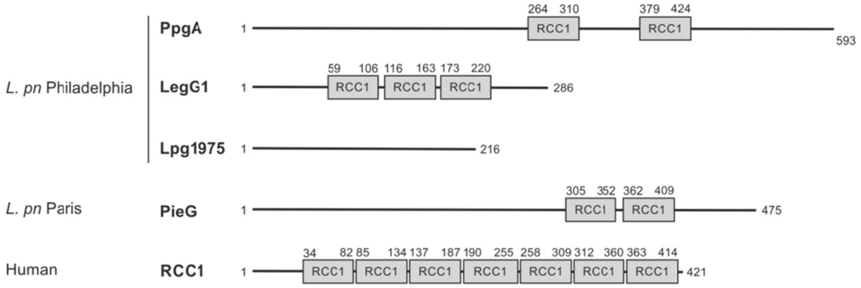

C

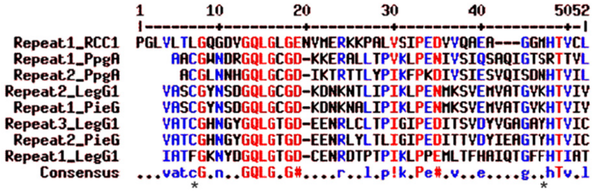

D

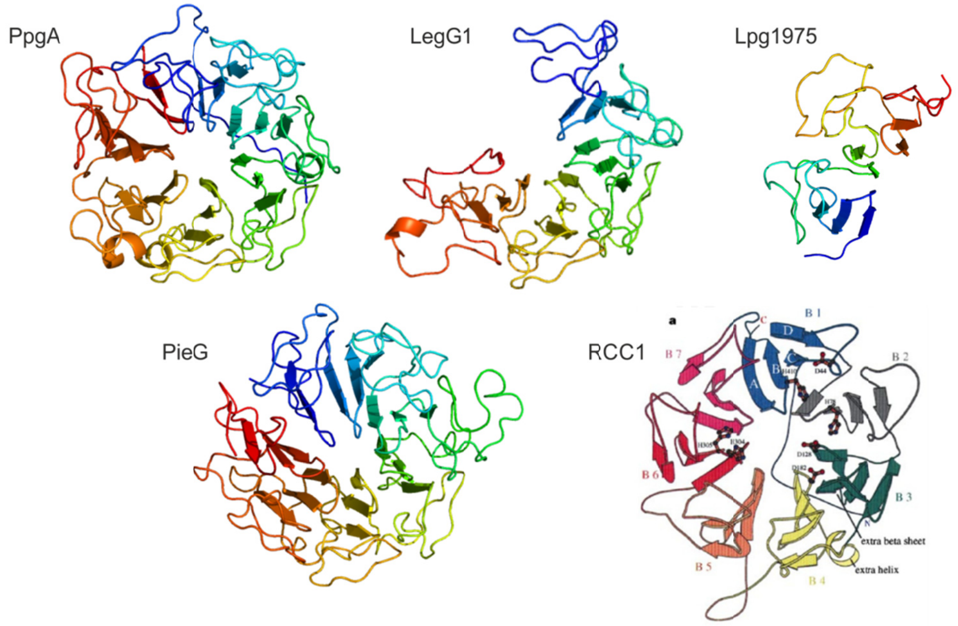

Fig. S2 (overleaf). Structural comparison of eukaryotic RCC1 and *L. pneumophila* RCC1 repeat effector proteins. (**A**) Amino acid sequences of the *L. pneumophila* protein PpgA (66 kDa), LegG1/Lpg1976 (31 kDa), Lpg1975 (25 kDa) and PieG (53 kDa). The RCC1 domains, which are predicted by the Pfam tool, are highlighted in red. (**B**) Schematic overview and position of RCC1 domains in *L. pneumophila* PpgA, LegG1, Lpg1975, PieG and the human Ran GEF RCC1. (**C**) Alignment of the RCC1 domains of PpgA, LegG1, and PieG with the first RCC1 domain of RCC1. Residues critical for GEF activity are marked with an asterisk (*) (Azuma et al., 1996). (**D**) Predicted structure of PpgA, LegG1, Lpg1975 and PieG (SWISS-MODEL) and comparison with the X-ray crystallography structure of human RCC1 forming a seven-bladed propeller (Renault et al., 1998).
