# Supplementary material for: Divergent Evolution of Legionella RCC1 Repeat Effectors Defines the Range of Ran GTPase Cycle Targets
Source: mBio. 2020 Mar 24;11(2):e00405-20. doi: 10.1128/mBio.00405-20 (PMC7157520; doi:10.1128/mBio.00405-20)
Supplement: FIG S3 [file mBio.00405-20-sf003.pdf]

Figure S3

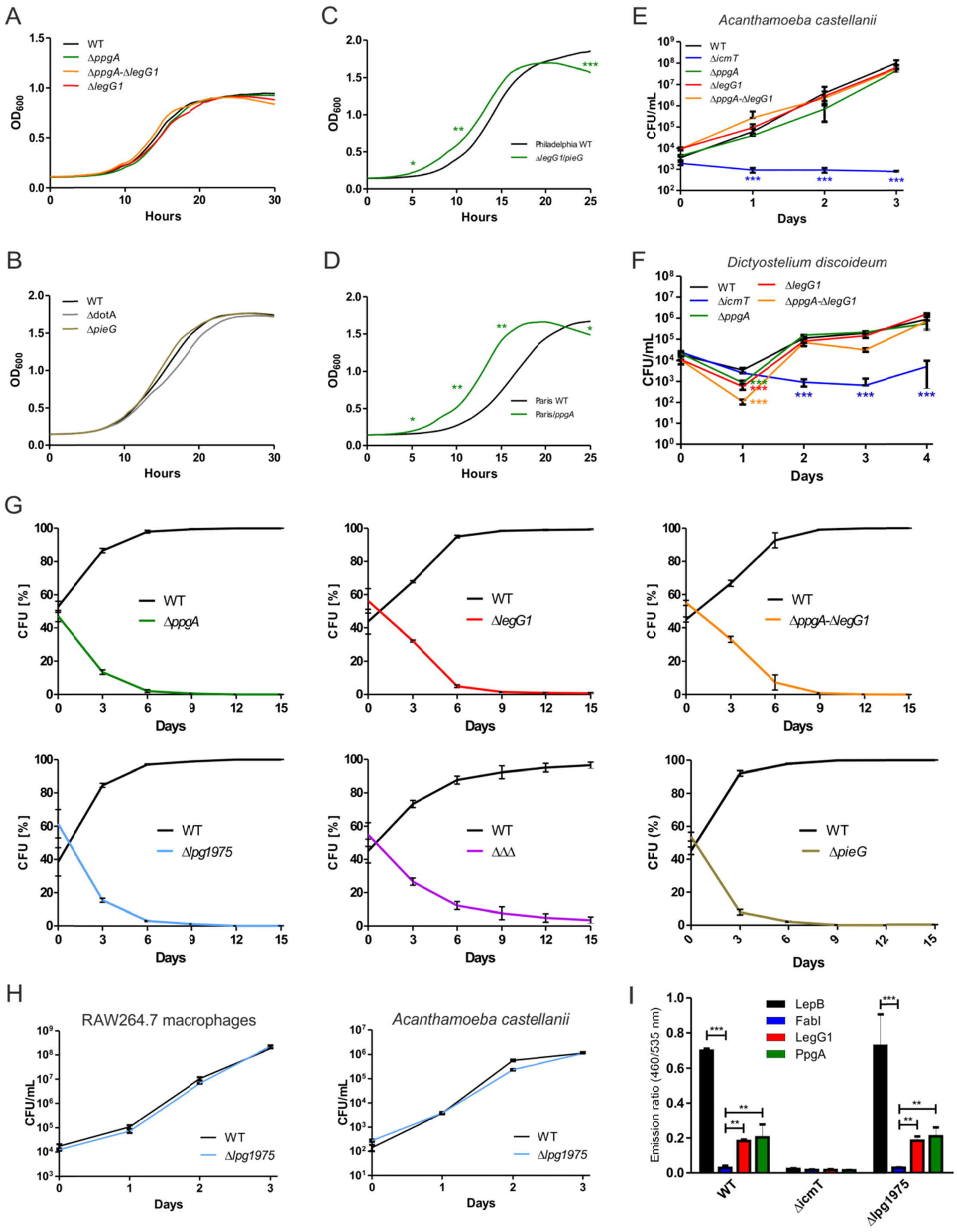

Fig. S3. Growth and competition of *L. pneumophila* strains lacking RCC1 repeat genes. Growth of *L. pneumophila* in AYE broth at 37°C, (**A**) strain JR32 or Δ*ppgA*, Δ*legG1*, Δ*ppgA*-Δ*legG1* mutants, (**B**) strain Paris or Δ*dotA*, Δ*pieG* mutants, (**C**) strain JR32 harboring vector (pCR033) or Δ*legG1* producing M45-PieG (pLS031), or (**D**) strain Paris harboring vector

(pCR033) or producing M45-PpgA (pLS006) (3 independent experiments; one-way ANOVA, *, *P*<0.1; **, *P*<0.01; ***, *P*<0.001). (**E**) *A. castellanii* or (**F**) *D. discoideum* was infected (MOI 0.1) with *L. pneumophila* strain JR32, Δ*icmT*, Δ*ppgA*, Δ*legG1*, or ΔppgA-Δ*legG1*, and intracellular replication at 30°C or 25°C, respectively, was assessed by CFU in lysates of the infected cells (3 independent experiments; two-way ANOVA, *, *P*<0.1; **, *P*<0.01; ***, *P*<0.001). (**G**) For competition assays, *A. castellanii* was co-infected at a 1:1 ratio (MOI of 0.01 each) with the *L. pneumophila* parental strain JR32 and Δ*ppgA* (LS03), Δ*legG1* (ER01), Δ*ppgA*-Δ*legG1* (LS01), Δ*lpg1975* (LS06) or Δ*ppgA*-Δ*legG1*-Δ*lpg1975* (LS05, ΔΔΔ), or with the parental strain Paris and Δ*pieG* (LS08) and grown at 37°C for 15 d. Every third day supernatant and lysed amoebae were diluted 1:1000, fresh amoebae were infected, and CFU were determined on agar plates containing kanamycin or not. (**H**) RAW 264.7 macrophages (left) or *A. castellanii* (right) were infected (MOI 0.1) with *L. pneumophila* strain JR32 or Δ*lpg1975*, and intracellular replication at 37°C or 30°C, respectively, was assessed by CFU. (**I**) RAW264.7 macrophages were infected (MOI 20, 1 h) with *L. pneumophila* wild-type strain JR32, Δ*icmT* or Δ*lpg1975* harboring pXDC61-*lepB*, pXDC61-*fabI*, pXDC61-*legG1* or pXDC61-*ppgA* (pLS044) encoding TEM β-lactamase fusion proteins. Enzymatic activity was assayed through hydrolysis of the fluorogenic substrate CCF4/AM (emission ratio 460/530 nm). Data show means and standard deviations of three independent experiments (Two-way ANOVA, **, *P*<0.01; ***, *P*<0.001).
