# Supplementary material for: Divergent Evolution of Legionella RCC1 Repeat Effectors Defines the Range of Ran GTPase Cycle Targets
Source: mBio. 2020 Mar 24;11(2):e00405-20. doi: 10.1128/mBio.00405-20 (PMC7157520; doi:10.1128/mBio.00405-20)
Supplement: TABLE S1 [file mBio.00405-20-st001.pdf]

Table S1A. Strains and plasmids used in this study.

Strain/plasmid	Relevant properties [a]	Reference
L. pneumophila		
DotA	Paris *dotA*::Kan^R (Δ*dotA*)	(Cazalet et al., 2004)
ER01	JR32 *lpg1976*::Kan^R (Δ*legG1*)	(Rothmeier et al., 2013)
GS3011	*L. pneumophila* JR32 *icmT3011*::Kan^R (Δ*icmT*)	(Segal and Shuman, 1998)
JR32	Virulent *L. pneumophila* sg 1 strain Philadelphia-1	(Sadosky et al., 1993)
LS01	JR32 *lpg2224*::Gen^R, *lpg1976*::Kan^R (Δ*ppgA*-Δ*legG1*)	This work
LS02	JR32 *lpg2224*::Gen^R (Δ*ppgA)*	This work
LS03	JR32 *lpg2224*::Kan^R (Δ*ppgA*)	This work
LS05	JR32 *lpg2224*::Gen^R, *lpg1976-1975*::Kan^R (ΔΔΔ)	This work
LS06	JR32 *lpg1975*::Kan^R (Δ*lpg1975*)	This work
LS08	Paris *pieG*::Kan^R (Δ*pieG*)	This work
Paris	Virulent *L. pneumophila* strain Paris	(Cazalet et al., 2004)
D. discoideum		
Ax3	Parental strain (lab collection)	(Loovers et al., 2007)
**Yeast**		
AH109	*MATa, trp1-901, leu2-3, 112, ura3-52, his3-200, gal4Δ, gal80Δ, LYS2 : : GAL1*$_{UAS}$*-GAL1*$_{TATA}$*-HIS3, GAL2*$_{UAS}$*-GAL2*$_{TATA}$*-ADE2, URA3 : : MEL1*$_{UAS}$*-MEL1*$_{TATA}$*-lacZ*	Clontech
BY474	*MATa*, *ura3 leu2 his3 met15 TRP1*	Euroscarf
prp20-1	*MATα*, *ura3 leu2 his3 prp20-1::TRP1*	(Schlenstedt et al., 1997)
rna1-1	*MATα*, *ura3 leu2 his3 rna1-1::TRP1*	(Schlenstedt et al., 1997)
yrb1-51	*NAT*, *ura3 leu2 his3 kan yrb1-51::TRP1*	(Künzler et al., 2001)
yrb2Δ	*MATa his3 leu2 met15 ura3 YRB2::KANMX*	Open Biosystems

Plasmids

pAW016	pDM323-calnexin (C'-GFP), G418^R, Amp^R	(Bärlocher et al., 2017)
pBSL141	oriR (pBM1), Amp^R, MCS::Gen^R	(Alexeyev et al., 1995)
pCaln-GFP	P_{act15}, calnexinA-RSSSKLK-GFP (S65T), G418^R, Amp^R	(Müller-Taubenberger et al., 2001)
pCR033	*Legionella* expression vector, Δ*mobA*, RBS, M45-(Gly)$_5$, Cam^R, (= pMMB207-C-RBS-M45)	(Urwyler et al., 2009)
pCR077	pMMB207C-P_{tac}-RBS-*dsred*-RBS-MCS, Cam^R	(Finsel et al., 2013)
pDM317	*Dictyostelium* expression vector, extra-chromosomal, N-terminal GFP, G418^R, Amp^R	(Veltman et al., 2009)
pEGFP-N1	Mammalian expression vector, EGFP N-terminal, Kan^R	Clontech
pER001	pDXA-EGFP-*RanBP1*, Amp^R	This work
pER005	pCR077-M45-*legG1*, Cam^R	(Rothmeier et al., 2013)
pER016	pDXA-EGFP-*legG1*$_{\Delta CAAX}$, Amp^R	This work
pER017	pDXA-EGFP-*legG1*, Amp^R	This work
pGBKT7	yeast expression vector, GAL4 act. dom., Amp^R	Takara Bio
pGADT7	yeast expression vector, GAL4 DNA-bd. dom., Amp^R	Takara Bio
pLAW344	*oriT* (RK2), *oriR* (ColE1), *sacB*, Cam^R, Amp^{R,} Kan^R	(Wiater et al., 1994)
pLS001	pLAW344-Δ*ppgA*::Gen^R	This work
pLS002	pLAW344-Δ*ppgA*::Kan^R	This work
pLS006	pMMB207-C-RBS-M45-*ppgA*, Cam^R	This work
pLS008	pCR077-M45-ppgA, Cam^R	This work
pLS023	pLAW344-Δ*lpg1975*:: Kan^R	This work
pLS024	pMMB207-C-RBS-M45-*lpg1975*-*legG1*, Cam^R	This work

pLS026	pCR077-M45- *lpg1975-legG1*, Cam^R	This work
pLS029	pLAW344-Δ*lpg1975_1976*:: Kan^R	This work
pLS031	pMMB207-C-RBS-M45-*pieG*, Cam^R	This work
pLS033	pCR077-M45-*pieG*, Cam^R	This work
pLS044	pMMB207C-*blaM-ppgA* (pXDC61), Cam^R	This work
pLS064	pLAW344-Δ*pieG*::Kan^R	This work
pLS078	pDM317-*ppgA* (N'-GFP), $G418^R$, Amp^R	This work
pLS084	YEP351gal-FLAG-*legG1*, Amp^R	This work
pLS085	YEP351gal-FLAG-*ppgA*, Amp^R	This work
pLS086	YEP351gal-FLAG-*pieG*, Amp^R	This work
pLS087	YEP351gal-FLAG-*lpg1975-legG1*, Amp^R	This work
pLS093	pEGFP-GFP-*pieG*, Kan^R	This work
pLS095	pEGFP-GFP-*ppgA*, Kan^R	This work
pLS102	pCR077-V5-*pieG*, Cam^R	This work
pLS106	pCR077-V5-*legG1*, Cam^R	This work
pLS109	pCR077-V5-*ppgA*, Cam^R	This work
pLS113	YEP351gal-GFP-*pieG*, Amp^R	This work
pLS118	YEP351gal-GFP-*legG1*, Amp^R	This work
pLS120	YEP351gal-GFP-*ppgA*, Amp^R	This work
pLS127	YEP352gal-*RNA1-1*-Strep, Amp^R	This work
pLS128	YEP352gal-*RNA1*-Strep, Amp^R	This work
pLS173	YEP352gal-Strep-*GSP1*, Amp^R	This work
pLS185	YEP352gal-Strep-*PRP20*, Amp^R	This work
pLS186	YEP352gal-Strep-*YRB1*, Amp^R	This work
pLS211	pGADT7-*legG1_33-286*, Amp^R	This work

pLS213	pGBKT7-*ranBP10*, Kan^R	This work
pLS226	pEGFP-Strep-*legG1*, Kan^R	This work
pLS227	pEGFP-GFP-*legG1*, Kan^R	This work
pLS229	pEGFP-Strep-*ppgA*, Kan^R	This work
pLS230	pEGFP-Strep-*pieG*, Kan^R	This work
pLS231	pEGFP-Strep-*rcc1*, Kan^R	This work
pLS233	pEGFP-GFP-*lpg1975*, Kan^R	This work
pLS234	pEGFP_GFP-*lpg1975-legG1*, Kan^R	This work
pLS235	pEGFP-GFP-$pieG_{189\text{-}475}$ (PieG_split) Kan^R	This work
pLS242	pEGFP-Strep-*ranBP10*, Kan^R	This work
pM1027	pGADT7-*pieG*, Amp^R	This work
pM1028	pGADT7-*ppgA*, Amp^R	This work
pM1057	pGBKT7-*ran*, Kan^R	This work
pM1058	pGBKT7-*ran* (Q69L), Kan^R	This work
pM1059	pGBKT7-*ran* (T24N), Kan^R	This work
pM1256	pGBKT7-*ran* (N122D), Kan^R	This work
pM1319	pET51b producing Strep-$LegG1_{33\text{-}286}$-His, Amp^R	This work
pM1593	pGBKT7-*ranGAP1*, Kan^R	This work
pMMB207C	*Legionella* expression vector, Δ*mobA*, no RBS, Cam^R	(Chen et al., 2004)
pNT28	pMMB207C-RBS-*gfp* (constitutive *gfp*), Cam^R	(Tiaden et al., 2007)
pRS316	URA1, CEN6, Amp^R	Addgene
pRS316-*RNA1*	URA1, CEN6, *RNA1*, Amp^R	(Yan et al., 1998)
pSW001	pMMB207-C, Δ$lacI^q$ (constitutive *dsred*), Cam^R	(Mampel et al., 2006)
pSW102	MCS-*gfp* in pDXA, $G418^R$, Amp^R	(Weber et al., 2009)
pUC4K	oriR (pBR322), Amp^R, MCS::Kan^R	Amersham

pWS034	pDM323-$P4C_{sidC}$-*gfp*, G418^R, Amp^R	(Welin et al., 2018)
pXDC61-FabI	pMMB207C-*blaM*-*fabI*, Cam^R	(de Felipe et al., 2008)
pXDC61-LegG1	pMMB207C-*blaM*-*legG1*, Cam^R	(de Felipe et al., 2008)
pXDC61-LepB	pMMB207C-*blaM*-*lepA*, Cam^R	(de Felipe et al., 2008)
pYEP351gal	LEU2, Amp^R	ATCC, 37672
pYEP352gal	URA3, Amp^R	ATCC, 37673

[a] Abbreviations: Amp, ampicillin; Cam, chloramphenicol; Kan, kanamycin; Gen, gentamicin.

Table S1B. Oligonucleotides used in this study.

**Oligo**	**Sequence (5'-3')**[a]	**Comment**
oER03	AGA CCA ATG TCG ACT CAT AAC AAA TTG CAT GGC G	3' of *legG1*, SalI
oER25	AAAAAATGCATTTGCATCTTGAATTGCATCAAATAAATGG	5' of *legG1*, NsiI
oER26	AAAAAATGCATTCATAACAAATTGCATGGCGAGAATTTAC	3' of *legG1*, NsiI
oER27	AAAAAATGCATTCATGGCGAGAATTTACTAATTTTCATC	3' of $legG1_{\Delta CAAX}$, NsiI
oLS001	AAAAACGCGTCGACTTGGCATGATGTTGATTTAG	5' of upstream region of *lpg1975* (deletion), SalI
oLS002	AAAAACGCGAATTCGATCTTTGGAATGGAAAAG	3' of upstream region of *lpg1975* (deletion), EcoRI
oLS003	AAAAACGCGAATTCTTGCATCTTGAATTGC	5' of downstream region of *lpg1975* (deletion), *EcoR*I
oLS004	AAAAACGCGTCGACTTCACCTTGTCCAGAAAC	3' of downstream region of *lpg1975* (deletion), SalI
oLS005	AAAAACGCGAATTCGGTTTAATTGGTCTCC	5' of downstream region of *lpg1976* (deletion), *EcoR*I
oLS006	AAAAACGCGTCGACTAAAAATCGCAAAACTGTC	3' of downstream region of *lpg1976* (deletion), SalI
oLS007	AAAAACGCTCTAGATAAAGCTACAGCCG	5' of upstream region of *ppgA*

		(deletion), XbaI
oLS008	AAAAACGCGTCGACGATATATATTTTCCATTAG	3' of upstream region of *ppgA* (deletion), SalI
oLS009	AAAAACGCGTCGACTGCAAAACATGCATTTTAG	5' of downstream region of *ppgA* (deletion), SalI
oLS010	AAAAACGCTCTAGATCCACCTCTCAAAATAAC	3' of downstream region of *ppgA* (deletion), XbaI
oLS011	AAAAACGCGGATCCGTGGGTTATAATGAAAC	5' of *lpg1975*, BamHI
oLS013	AAAAACGCCCCGGGGAATGGATCGGAGTAGG	5' of M45, SmaI
oLS014	AAAAACGCGGATCCATGCCTGATGTTAGC	5' of *pieG*, BamHI
oLS015	AAAAACGCGTCGACTCATAGCAAATTACATGG	3' of *pieG*, SalI
oLS016	AAAAACGCGGATCCATGAAAGAACCTCG	5' of *ppgA*, BamHI
oLS017	AAAAACGCGTCGACTTAGGCGCTTCGTTTGG	3' of *ppgA*, SalI
oLS018	CAACCTCATGCAAGTGGACAAACGCTTAAAT	silent mutation to remove SalI from *ppgA* ORF, 5'
oLS019	ATTTAAGCGTTTGTCCACTTGCATGAGGTTG	silent mutation to remove SalI from *ppgA* ORF, 3'
oLS043	ATATTTTAAACCCTATTTTTCTCTTTTCCCG	5' within *lpg1975* ORF, insertion of T to fuse *lpg1975-legG1* (*lpg1976*)
oLS044	CGGGAAAAGAGAAAAATAGGGTTTAAAATAT	3' within *lpg1975* ORF, insertion of T to fuse *lpg1975-legG1* (*lpg1976*)
oLS073	AAAAACGCGGATCCCAACACAGGCAACGAC	5' of upstream region of *pieG* (deletion), BamHI
oLS074	AAAAACGCCTGCAGAACGGTTGGTGACAGATG	3' of upstream region of *pieG* (deletion), PstI
oLS075	TTTTTCGCCTGCAGTCATACTGTTATTTGTGG	5' of downstream region of *pieG* (deletion), *Pst*I
oLS076	AAAAACGCGGATCCGCAAAATGATTATTCCTC	3' of downstream region of *pieG* (deletion), BamHI
oLS079	TTTTTCGCAAGCTTTTAGGCGCTTCGTTTGG	3' of *ppgA*, HindIII

oLS104	AAAAAGCGAGATCTAAAATGAAAGAACCTCGTG	5' of *ppgA*, BglII
oLS105	TTTTTCGCACTAGTGGCGCTTCGTTTGGG	3' of *ppgA*, SpeI
oLS123	AAAAACGC*GGATCC*ATGGACTACAAGGACGACGATGACAA GCCTGATGTTAGCGGC	5' of *pieG* (N'FLAG), BamHI
oLS124	AAAAACGCGGATCCATGGACTACAAGGACGACGATGACAA GAAAGAACCTCGTGAAC	5' of *ppgA* (N'FLAG), BamHI
oLS125	AAAAACGCGGATCCATGGACTACAAGGACGACGATGACAA GCATCTTGAATTGCATC	5' of *legG1* (N'FLAG), BamHI
oLS133	AAAAAGCGGTCGACATGAAAGAACCTCGTG	5' *ppgA*, SalI
oLS134	AAAAACGCGGATCCTTAGGCGCTTCGTTTGG	3' *ppgA*, BamHI
oLS135	AAAAAGCGGTCGACATGCCTGATGTTAGCGG	5' *pieG*, SalI
oLS136	AAAAACGCGGATCCTCATAGCAAATTACATGG	3' *pieG*, BamHI
oLS174	AAAAACGCGGATCCATGGGTAAAGGAGAAG	5' GFP, BamHI
oLS185	AAAAACGCGTCGACATGGCTACCTTGCACTTCG	5' *RNA1*, SalI
oLS186	TTTTTCGCCTGCAGTTACTTCTCGAACTGAGGATGCGACCA AGATCTTTTGATTTCAGTTTCAGC	3' *RNA1* (C'-Strep), PstI (BglII)
oLS216	TTTTTCGCGCGGCCGCTTAGGCGCTTCGTTTGGG	3' *ppgA*, NotI
oLS230	AAAAACGCCTCGAGTCATAACAAATTGCATG	3' *legG1*, XhoI
oLS236	TTTTTGCGGTCGACATGTTGCATCTTGAATTGC	5' *legG1*, SalI
oLS238	AAAAACGCGGATCCTCATAACAAATTGCATG	3' *legG1*, BamHI
oLS250	AAAAAGCGAGATCTAAAATGGCGGCGGCGACGGCAG	5' *ranBP10*, BglII
oLS251	TTTTTCGCAAGCTTCTAGTGCAAGTAGTCATCG	3' *ranBP10*, HindIII
oLS259	TTTTTCGCCTCGAGCTAGTGCAAGTAGTCATC	3' *ranBP10*, XhoI
oLS265	AAAAACGCGCGGCCGCTCATAGCAAATTACATGG	3' *pieG*, NotI
oLS279	AAAAAGCGAGATCTAAAATGTCTGCCCCAGCTGC	5' *GSP1*, BglII
oLS280	TTTTTCGCAAGCTTTTATAAATCAGCATCATC	3' *GSP1*, HindIII
oLS281	AAAAAGCGAGATCTAAAATGTCTAGCGAAGATAAG	5' *YRB1*, BglII
oLS282	TTTTTCGCAAGCTTCTAAGCCTTTTTGTTGATTTC	3' *YRB1*, HindIII
oLS285	AAAAAGCGAGATCTAAAATGGTCAAAAGAACAGTCG	5' *PRP20*, BglII
oLS286	TTTTTCGCAAGCTTTTAATCATCCATTTCATCC	3' *PRP20*, HindIII

oLS318	AAAAACGCGGATCCCTAGTGCAAGTAGTCATC	3' *ranBP10*, BamHI
oLS327	AAAAACGCCATATGAAAACAGTAAAACAAATACATTC	5' *legG1*$_{33}$, NdeI
oLS328	AAAAAGCGCATATGGCGGCGGCGACGGCAGAC	5' *ranBP10*, NdeI
oLS331	AAAAAGCGCATATGCCTGATGTTAGCGGC	5' *pieG*, NdeI
oLS332	AAAAACGCGGATCCTCATAGCAAATTACATGG	3' *pieG*, BamHI
oLS346	AAAAAGCGAAGCTTATGTGGTCGCATCCTCAGTTCGAGAA GGATATCTTGCATCTTGAATTGCATC	5' *legG1* (N' Strep), HindIII (EcoRV)
oLS347	AAAAACGCGCGGCCGCTCATAACAAATTGCATGG	3' *legG1*, NotI
oLS350	AAAAAGCGGATATCAAAGAACCTCGTGAACTTAATCG	5' *ppgA*, EcoRV
oLS351	AAAAAGCGGATATCCCTGATGTTAGCGGCAAACC	5' *pieG*, EcoRV
oLS352	AAAAAGCGGATATCTCACCCAAGCGCATAGC	5' *rcc1*, EcoRV
oLS353	TTTTTCGCGCGGCCGCTCAGCTCTGTTCTTTGTCC	3' *rcc1*, NotI
oLS358	AAAAAGCGGTCGACGTGGGTTATAATGAAACC	5' *lpg1975*, SalI
oLS359	AAAAACGCGGATCCTTATTTGATGCAATTCAAG	3' *lpg1975*, BamHI
oLS363	AAAAAGCGGTCGACTTGAGTCTTCAATTACATAAAATAA	5' *pieG*$_{189}$, SalI
oLS364	AAAAACGCGGATCCTCATAGCAAATTACATGG	3' *pieG*, BamHI
oLS365	AAAAAGCGGATATCATGGCGGCGGCGACGGC	5' r*anBP10*, EcoRV
oLS366	AAAAACGCGCGGCCGCCTAGTGCAAGTAGTCATC	3' *ranBP10*, NotI
o51	CCCCATATGAAAACAGTAAAACAAATACATTCA	5' *legG1*, NdeI
o1411	ATCTGCAGCTCGAGTCATGGCGAGAATTTACTAA	3' *legG1*, XhoI
o1936	CAGATTACGCTCATATGGCGGCGGCGACGGCAGA	5' *ranBP10*, NdeI
o1937	TCGAGCTCGATGGATCCCTAGTGCAAGTAGTCAT	3' *ranBP10*, BamHI

Table S1C. Oligonucleotides used for RNA interference.

NCBI gene	Gene description	Entrez Gene ID	Product name	Product ID
Unspecific_AllStars_1	AllStars	0	Unspecific_AllStars_1	SI03650318
ARF1	ADP-ribosylation factor 1	375	Hs_ARF1_1 (1)	SI00299250
RanGAP1	Ran GTPase activating protein 1	3905	Hs_RANGAP1_4	SI00698376
RanGAP1	Ran GTPase activating protein 1	3905	Hs_RANGAP1_6	SI04146184
RanGAP1	Ran GTPase activating protein 1	3905	Hs_RANGAP1_7	SI04289810
RanGAP1	Ran GTPase activating protein 1	3905	Hs_RANGAP1_8	SI04293751
RanBP10	Ran-binding protein 10	57610	Hs_RANBP10_3	SI00128079
RanBP10	Ran-binding protein 10	57610	Hs_RANBP10_6	SI02644355
RanBP10	Ran-binding protein 10	57610	Hs_RANBP10_7	SI02644362
RanBP10	Ran-binding protein 10	57610	Hs_RANBP10_9	SI03114874
