# Supplementary material for: Divergent Evolution of Legionella RCC1 Repeat Effectors Defines the Range of Ran GTPase Cycle Targets
Source: mBio. 2020 Mar 24;11(2):e00405-20. doi: 10.1128/mBio.00405-20 (PMC7157520; doi:10.1128/mBio.00405-20)
Supplement: FIG S4 [file mBio.00405-20-sf004.pdf]

Figure S4

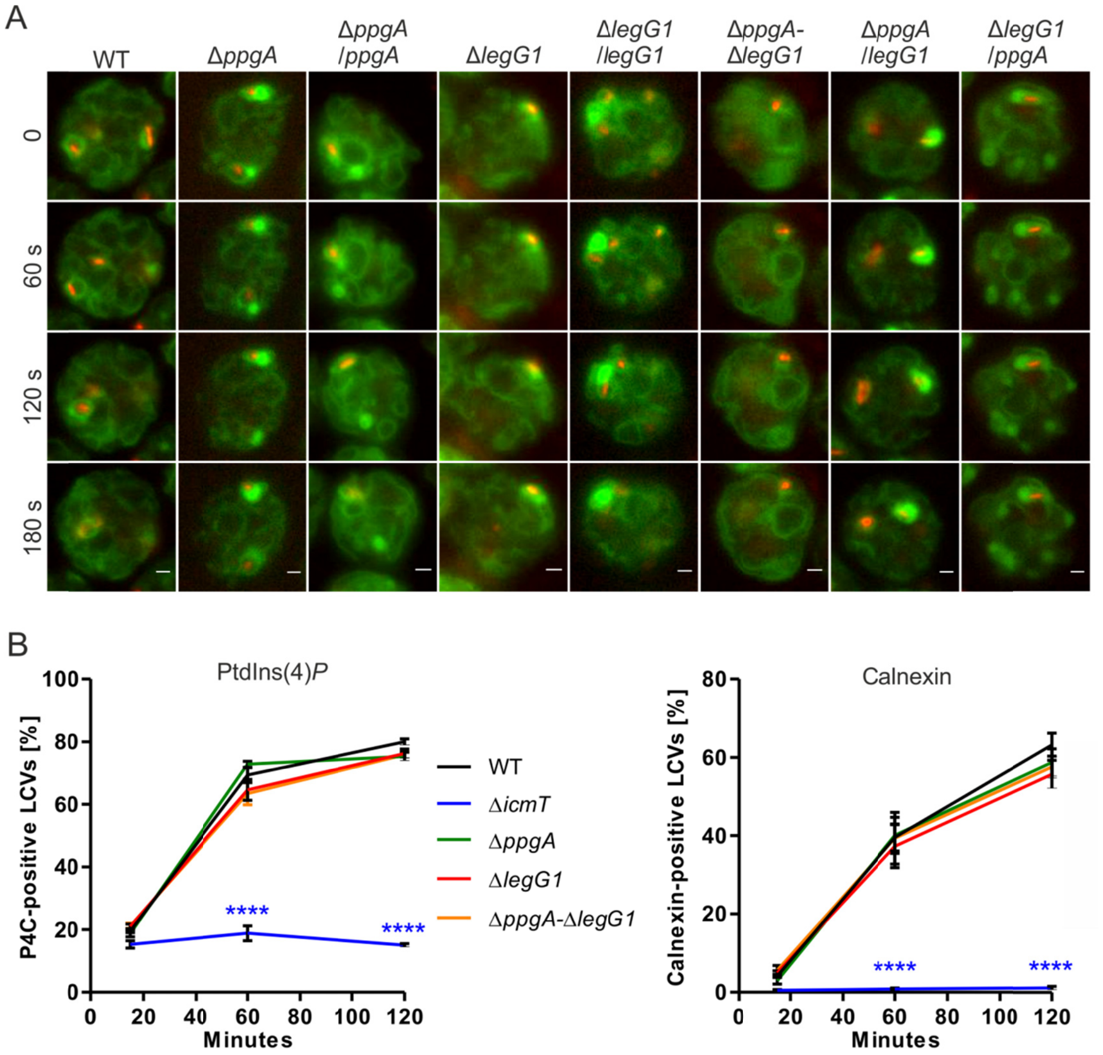

Fig. S4. PpgA controls LCV motility. (**A**) Real-time fluorescence microscopy of LCV motility in *D. discoideum* producing calnexin-GFP (pCaln-GFP) (green) infected (MOI 5, 1 h) with *L. pneumophila* JR32, Δ*ppgA*, Δ*legG1*, or Δ*ppgA*-Δ*legG1* producing DsRed (pCR077), DsRed and M45-LegG1 (pER005), or DsRed and M45-PpgA (pLS008) (red). 1-2 h p.i., LCV motility was recorded by laser confocal scanning microscopy for 180 s with images taken every 10 s. Figure shows representative images taken every 60 s for the strains indicated. Bars, 1 µm. (**B**) *D. discoideum* Ax3 producing the PtdIns(4)*P* probe $P4C_{SidC}$-GFP (pWS34; left) or calnexin-GFP (pAW016; right) was infected (MOI 5; 15, 60 or 120 min) with DsRed-producing *L. pneumophila* JR32, Δ*icmT*, Δ*ppgA*, Δ*legG1* or Δ*ppgA*-Δ*legG1* (pCR077), and based on the IFC co-localization scores the percentages of cells containing a GFP-positive LCV were quantified (means and SEMs from three independent experiments are shown; two-way ANOVA, ****, *P*<0.0001).
