# Supplementary material for: Divergent Evolution of Legionella RCC1 Repeat Effectors Defines the Range of Ran GTPase Cycle Targets
Source: mBio. 2020 Mar 24;11(2):e00405-20. doi: 10.1128/mBio.00405-20 (PMC7157520; doi:10.1128/mBio.00405-20)
Supplement: FIG S5 [file mBio.00405-20-sf005.pdf]

Figure S5

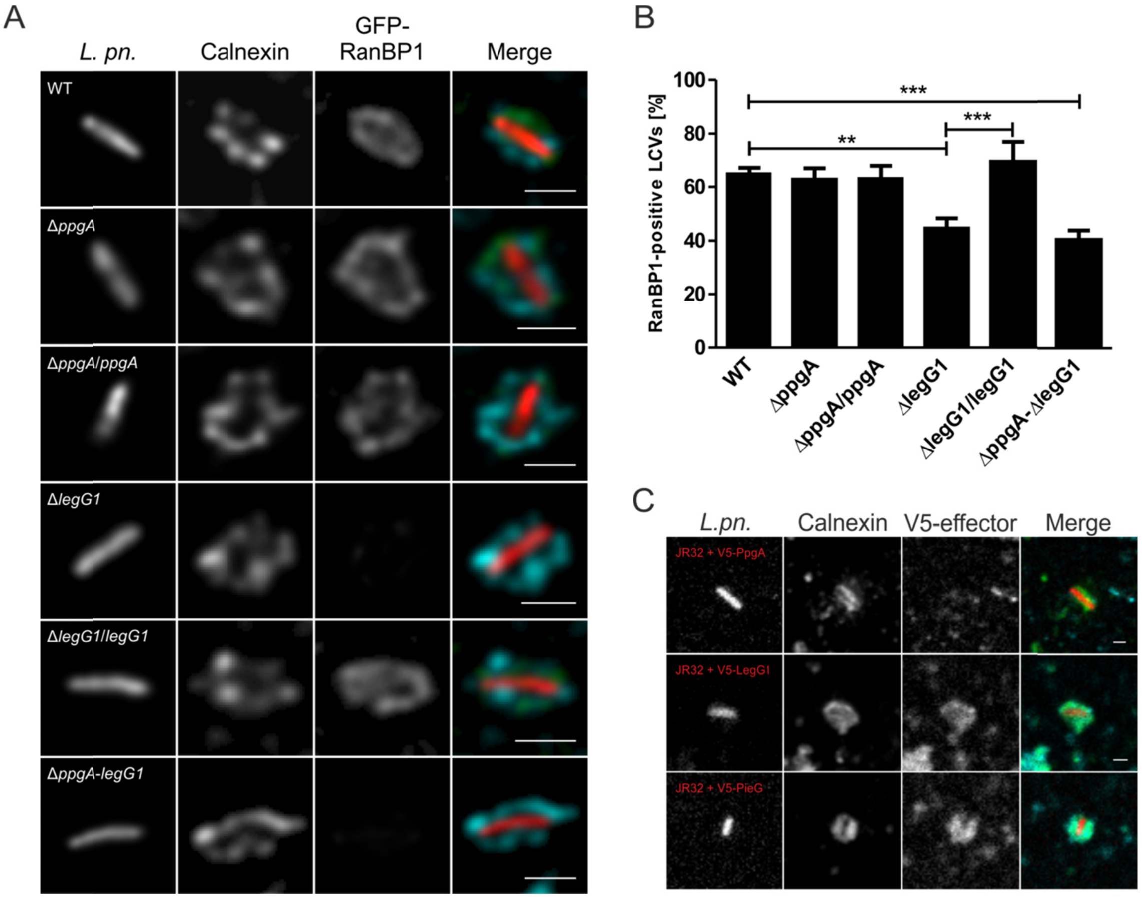

Fig. S5. Ran activation on LCVs and membrane localization of RCC1 repeat effectors. (**A**) *D. discoideum* producing GFP-RanBP1 (pER001) (green) was infected (MOI 30, 1 h) with *L. pneumophila* JR32, Δ*ppgA*, Δ*legG1*, or Δ*ppgA*-Δ*legG1* producing DsRed (pCR077), DsRed and M45-LegG1 (pER005), or DsRed and M45-PpgA (pLS008) (red), LCVs were isolated and immunostained for calnexin (blue), and localization of RanBP1 to LCVs was analyzed by confocal microscopy. Bars, 1 µm. (**B**) The percentage of GFP-RanBP1-positive LCVs (A) was scored (n = 100/strain, 3 independent experiments; One-way ANOVA, **, *P*<0.01; ***, *P*<0.001). (**C**) *D. discoideum* Ax3 infected (MOI 10, 2 h) with *L. pneumophila* JR32 producing DsRed and V5-PpgA (pLS109), V5-LegG1 (pLS106) or V5-PieG (pLS102). 1-2 h p.i. the amoebae were lysed, immunostained for calnexin (green) and V5 (cyan), and the localization of RCC1 effectors on LCVs was analyzed by confocal microscopy. Bars, 1 µm.
