# Supplementary material for: Divergent Evolution of Legionella RCC1 Repeat Effectors Defines the Range of Ran GTPase Cycle Targets
Source: mBio. 2020 Mar 24;11(2):e00405-20. doi: 10.1128/mBio.00405-20 (PMC7157520; doi:10.1128/mBio.00405-20)
Supplement: FIG S6 [file mBio.00405-20-sf006.pdf]

Figure S6

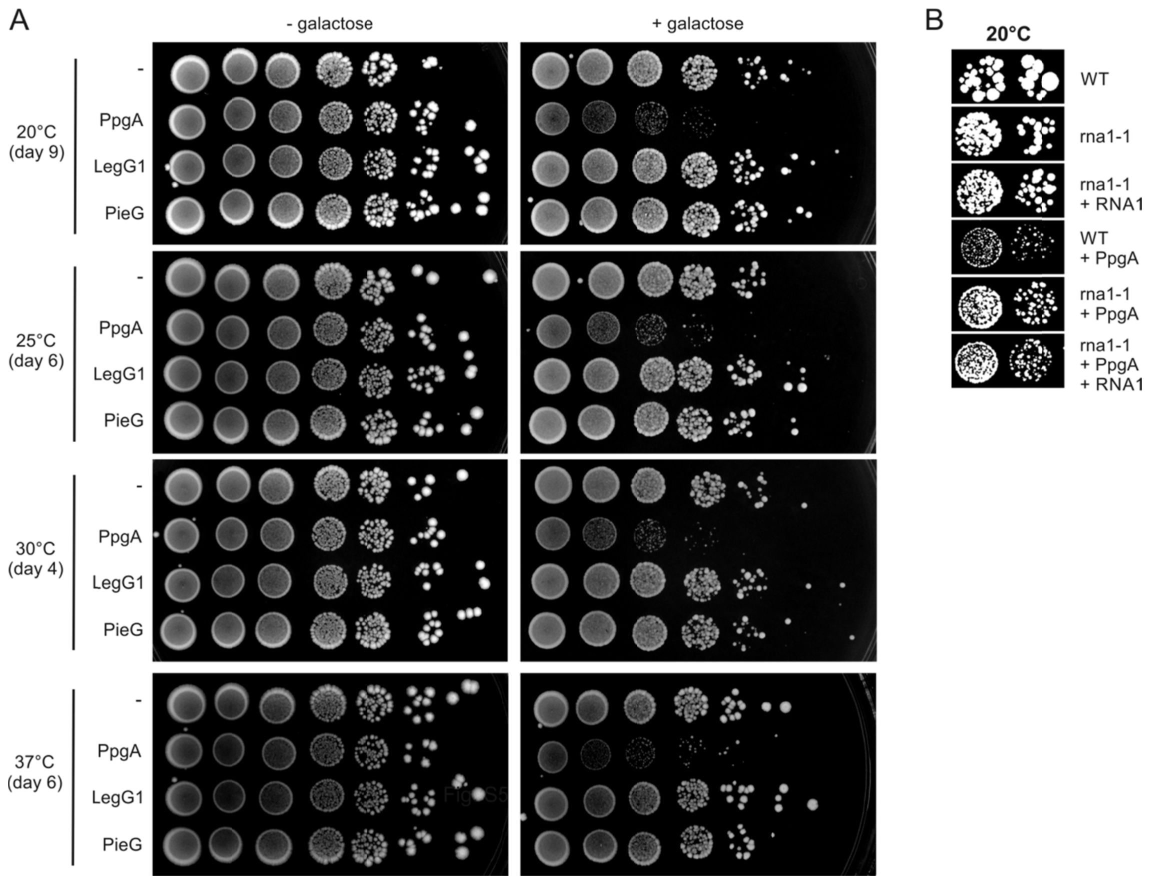

Fig. S6. *S. cerevisiae* dot spot controls and mutant complementation. (**A**) The *S. cerevisiae* mutant strains *rna1-1*, *prp20-1*, *yrb1-51* or *yrb2Δ* containing an empty plasmid (pYEP351gal) or a plasmid producing FLAG-PpgA (pLS085), FLAG-LegG1 (pLS084) or FLAG-PieG (pLS086) were spotted in 10-fold dilutions on SD-plates (- galactose, left panel) or SG-plates (+ galactose, right panel) without leucine and grown at the indicated temperatures for 5 (30°C), 6 (25°C and 37°C) or 7 (20°C) days. (**B**) *S. cerevisiae* BY4741 or *rna1-1* mutant containing the empty plasmids pYEP351gal and pRS316, pYEP351gal and pRS316-*RNA1* (RNA1), pRS316 and pLS085 (FLAG-PpgA), or pRS316-*RNA1* (RNA1) and pLS085 (FLAG-PpgA) were spotted in 10-fold dilutions on SG-plates without leucine and uracil and grown at the indicated temperatures for 3-6 days.
