# Supplementary material for: Divergent Evolution of Legionella RCC1 Repeat Effectors Defines the Range of Ran GTPase Cycle Targets
Source: mBio. 2020 Mar 24;11(2):e00405-20. doi: 10.1128/mBio.00405-20 (PMC7157520; doi:10.1128/mBio.00405-20)
Supplement: FIG S7 [file mBio.00405-20-sf007.pdf]

Figure S7

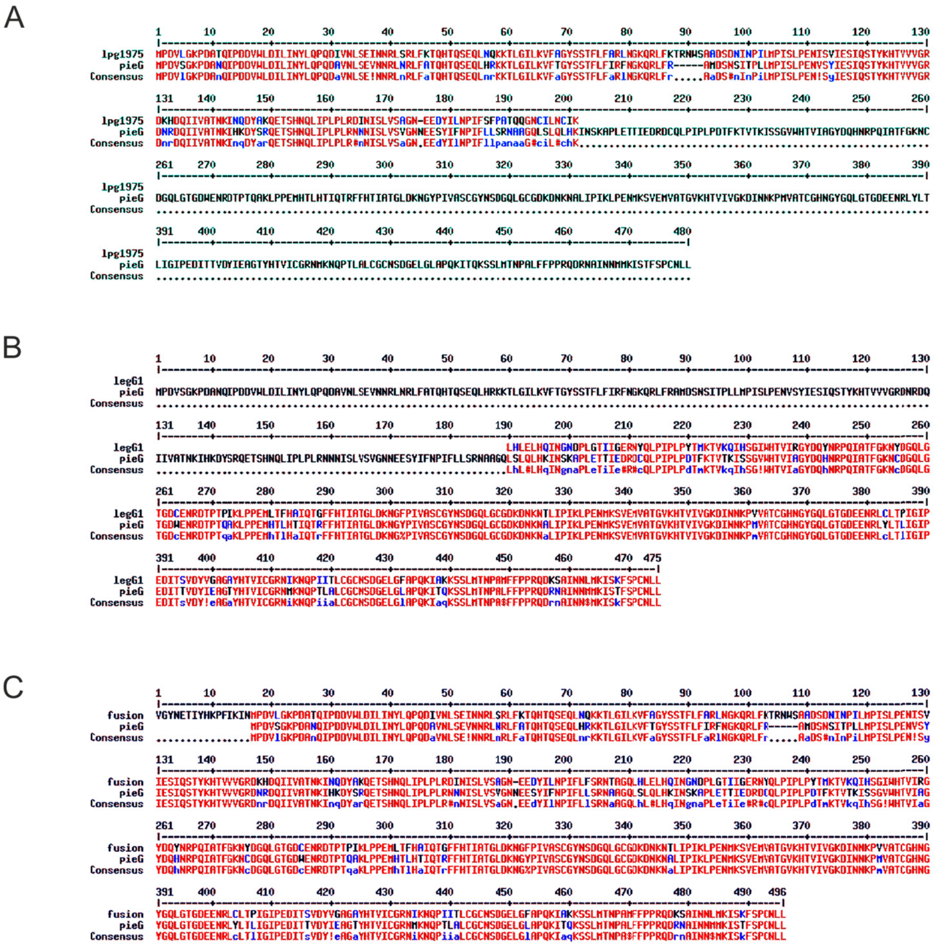

Fig. S7. Alignment of *L. pneumophila* RCC1 repeat genes. The *L. pneumophila* RCC1 repeat genes *lpg1975*, *legG1* and *pieG* were aligned using MultAlin. Alignment of (**A**) *pieG* with *lpg1975*, (**B**) *pieG* with *legG1* and (**C**) *pieG* with the fusion of *lpg1975* and *legG1*.
