# Supplementary material for: Divergent Evolution of Legionella RCC1 Repeat Effectors Defines the Range of Ran GTPase Cycle Targets
Source: mBio. 2020 Mar 24;11(2):e00405-20. doi: 10.1128/mBio.00405-20 (PMC7157520; doi:10.1128/mBio.00405-20)
Supplement: TEXT S1 [file mBio.00405-20-s0001.pdf]

Supplementary Methods and References

Bioinformatics analysis

All 59 *L. pneumophila* genomes from genbank (NCBI database) with a complete genome sequenced and available proteomes were downloaded. For the identification of RCC1 repeat proteins in *L. pneumophila* genomes the Pfam tool was used (Mitchell et al., 2015). A phylogenetic tree of these strains was inferred using Parsnp, a fast core-genome multi-aligner (Treangen et al. 2014). In parallel the same tree but adding the outgroup species *L. norrlandica* was reconstructed also with Parsnp to be able to root the *L. pneumophila* strains tree. Protein structures were predicted using SWISS-MODEL (Waterhouse et al., 2018). Alignment of protein sequences was performed with MultAlin.

The orthologous relationship among the proteins of all the selected strains was obtained running OrthoMCL (Li et al., 2003) with an inflation index of 2.5. Based on the OrthoMCL results, the presence/absence of genes orthologs of PieG (Lpp1959) and PpgA (Lpg2224) was represented besides the strains phylogeny. Cases were the corresponding genes were duplicated-triplicated or unexpectedly absent were verified manually by blast. Finally, gene trees of Lpp1959 and Lpg2224 were done to compare the protein trees versus the strains trees. With this aim protein alignments of these proteins were carried out using Muscle (Edgar, 2004) and tree reconstruction was done using neighbor-joining using as a model kimura 2 parameters and 100 bootstrap replicates. Final obtained trees were editing using iTOL (Letunic and Bork, 2007).

Statistics were performed using GraphPad Prism.

Bacteria and cells: growth conditions, infection and transformation

L. pneumophila strains (Table S1A) were grown on CYE agar plates, containing the corresponding antibiotic, for 3 days at 37°C. For infection, liquid cultures in ACES yeast extract (AYE) medium were inoculated at an OD_{600} of 0.1 and grown at 37°C for 21-22 h to

an early stationary phase (~2×10^9 bacteria/ml). Chloramphenicol (Cam; 5 μg/ml) and isopropyl-β-D-thiogalactopyranoside (IPTG; 1 mM) were added as required. Cultures were diluted to desired density (MOI 0.1-50), infection of phagocytes was synchronized by centrifugation (450 *g*, 10 min RT), and infected cells were incubated at the temperature and for the time indicated. *E. coli* TOP10 (Invitrogen) was cultured in LB broth, and antibiotics were added as required at the following concentrations: Cam (30 μg/ml), kanamycin (Kan; 50 μg/ml) or ampicillin (Amp; 100 μg/ml).

S. cerevisiae strains (Table S1A) were grown at various temperatures in complemented yeast nitrogen base (ForMedium) lacking uracil (Ura) and/or leucine (Leu) when necessary. Preparation of media, cultivation and yeast transformations were performed according to established procedures. All yeast plasmids used contained a *GAL1* promoter and were induced by supplementing growth media with 2% galactose.

D. discoideum Ax3 amoebae were grown in HL-5 medium (ForMedium) at 23°C and transfected by electroporation with a Gene Pulser Xcell (BioRad) device as described (Weber and Hilbi, 2014; Weber et al., 2014). After 24 h, transfectants were selected and maintained in HL5 medium containing geneticin (G418; 20 μg/ml). Murine macrophage-like RAW 264.7 cells and human A549 lung epithelial carcinoma cells were cultivated in RPMI 1640 medium (Life Technologies) supplemented with 10% heat-inactivated fetal bovine serum (FBS; Life Technologies) and 1% glutamine (Life Technologies). The cells were cultured at 37°C with 5% CO_2 in a humidified atmosphere. HEK 293T cells were cultivated in DMEM 1640 medium (Life Technologies) supplemented with 10% heat-inactivated fetal bovine serum (FBS; Life Technologies) and 1% glutamine (Life Technologies). The cells were cultured at 37°C with 5% CO_2 in a humidified atmosphere.

Molecular cloning and construction of *L. pneumophila* mutant strains

Plasmids and oligonucleotides used in this study are listed in Table S1A and Table S1B, respectively. DNA manipulations were performed according to standard protocols, and plasmids were isolated using commercially available kits from Macherey-Nagel or Qiagen.

Chromosomal deletion of *ppgA, lpg1975*, the double deletion of *legG1* and *ppgA*, the triple deletion of *lpg1975*, *legG1* and *ppgA* and the deletion of *pieG* were performed as previously described (Tiaden et al., 2007). In short, 1000 bp upstream and downstream fragments of the gene of interest were PCR amplified using DNA of *L. pneumophila* strain Philadelphia as a template for *lpg1975*, *lpg1975-lpg1976* and *ppgA* and DNA of *L. pneumophila* strain Paris for *pieG*. Primers oLS001/oLS002 and oLS003/oLS004 were used

for the respective *lpg1975* flanking regions, primers oLS005/oLS006 for the right flanking region of *lpg1976*, primers oLS007/oLS008 and oLS009/oLS010 for the *ppgA* flanking regions and primers oLS073/oLS074 and oLS075/oLS076 for the *pieG* flanking regions. The up- and downstream fragments together with a Kan^R resistance cassette were directly inserted into the pLAW344 suicide plasmid by a four way ligation. For the *ppgA* deletion plasmid, pLS002, the fragments were ligated into pLAW344 with XbaI and SalI for the Kan^R cassette. For the construction of the Δ*legG1*-Δ*ppgA* double mutant a similar plasmid was cloned containing a gentamicin resistance (Gen^R) cassette instead of Kan^R. For the *lpg1975* deletion plasmid, pLS023, the fragments were ligated using SalI and EcoRI for the Kan^R cassette and for the *pieG* deletion plasmid, pLS064, the fragments were ligated using BamHI and PstII for the Kan^R cassette. Clones were confirmed by sequencing, transformed by electroporation into *L. pneumophila* Philadelphia-1 JR32 for the *ppgA* and *lpg1975* mutant strains, ER01 (Δ*legG1*) for the double mutant, or *L. pneumophila* Paris for the *pieG* mutant strain, and selected for Kan^R or Gen^R and Suc^R colonies.

Translational M45-fusion proteins of *ppgA* were constructed by PCR amplification of *L. pneumophila* Philadelphia DNA or pLS006 using the primer pairs oLS016/oLS017 and oLS013/oLS017, respectively. Primers oLS018 and oLS019 were used to remove SalI from the *ppgA* ORF. The PCR products were digested with the BamHI/SalI and inserted into pMMB207C-RBS-M45 (pCR033) or pMMB207C-RBS-DsRed-RBS (pCR077), yielding pLS006 or pLS008, respectively. Moreover, *ppgA* was amplified from DNA of *L. pneumophila* JR32 using the primers oLS016 and oLS079, and inserted into pXDC61 via restriction sites BamHI/HindIII to obtain pLS044, encoding a TEM fusion protein.

Translational M45-fusion proteins of *lpg1975-legG1*_fusion were constructed by PCR amplification of *L. pneumophila* Philadelphia-1 DNA or pLS024 using the primer pairs oLS011/oER03 and oLS013/oER03, respectively. The primers oLS043 and oLS044 were used to add an additional thymidine phosphate to fuse the *lpg1975* and *legG1* ORFs. The PCR products were digested with the BamHI/SalI and inserted into pMMB207C-RBS-M45 (pCR033) or pMMB207C-RBS-DsRed-RBS (pCR077), yielding pLS024 or pLS026, respectively.

Translational M45-fusion proteins of *pieG* were constructed by PCR amplification of *L. pneumophila* Paris DNA or pLS031 using the primer pairs oLS014/oLS015 and oLS013/oLS015, respectively. The PCR products were digested with the BamHI/SalI and inserted into pMMB207C-RBS-M45 (pCR033) or pMMB207C-RBS-DsRed-RBS (pCR077), yielding pLS031 or pLS033, respectively.

D. discoideum expression plasmids were constructed by PCR amplification of the corresponding genes using oligonucleotide pairs oLS104/oLS105 (*ppgA*), oER025/oER026 (*legG1*) or oER025/oER027 (*legG1*$_{\Delta CAAX}$). Products were cut with the appropriate enzymes, and *ppgA* was ligated into pDM317 (pLS078), whereas *legG1* and *legG1*$_{\Delta CAAX}$ were inserted into pSW002, yielding pER17 and pER16, respectively.

For the production of FLAG-tagged *L. pneumophila* effectors in yeast, the corresponding genes were amplified with the primers oLS125/oER03, oLS124/oLS017, oLS123/oLS015 or oLS126/oER03, and inserted into pYEP351gal using BamHI/SalI yielding the plasmids pLS084, pLS085, pLS086 and pLS087, respectively. For the construction of pLS087 primers oLS043 and oLS044 were used to insert an additional nucleotide (thymidine phosphate) into the *lpg1975* ORF to obtain a continuous ORF, fusing *lpg1975* and *legG1* (*lpg1976*). Yeast plasmids encoding N'-terminal GFP fusions were constructed by amplification of the corresponding genes with the primers oLS174/oLS015, oLS174/oER03 or oLS174/oLS017. Ligation into pYEP351gal using the restriction sites BamHI/SalI resulted in the plasmids pLS113, pLS118 and pLS120, respectively. For the construction of yeast plasmids containing Strep-tagged fusion proteins, first, pLS128 or pLS127 (C-terminal Strep-tagged RNA1 or RNA1-1) were cloned, containing an additional BglII restriction site for subsequent cloning. Using the primers oLS185/oLS186 *RNA1* or *RNA1-1* was amplified from yeast genomic DNA (BY4741 or *rna1-1*), and the PCR products were subsequently inserted into pYEP352gal using SalI/PstI. pLS173, pLS174, pLS185 and pLS186 were constructed by amplification of the corresponding genes from yeast genomic DNA (BY4741), for *GSP1*, *PRP20* and *YRB1* or pLS212, for *ranBP10*, using the primer pairs oLS279/oLS280, oLS285/oLS286, oLS281/oLS282 or oLS250/oLS251, respectively. The PCR products obtained were inserted into the backbone of pLS137, which was digested with BglII/HindIII, resulting in plasmids for the production of N'-terminally Strep-tagged fusion proteins in yeast.

For the yeast two-hybrid screening, *legG1*$_{33\text{-}287}$, *ppgA*, *pieG* or *RanBP10* were cloned into the prey vector pGADT7 and the bait vector pGBKT7. Using primers oLS327/oLS230, o1411/o51 or oLS328/oLS259, the respective genes were amplified and inserted into pGADT7 using NdeI/XhoI as restriction sites. The primers o1411/o51, oLS331/oLS332 or oLS328/oLS318 were used to amplify the respective genes for cloning into the pGBKT7 backbone using the restriction sites NdeI/XhoI for *legG1*$_{33\text{-}287}$ and NdeI/PstI for *ppgA*, or NdeI/BamHI, for *pieG* and *ranBP10*.

Mammalian expression plasmids encoding Strep-fusion proteins were cloned as following: *Strep-legG1* was amplified using primers oLS346/oLS347 from *L. pneumophila Philadelphia* genomic DNA. The backbone, pEGFP-N1 was linearized with HindIII/NotI (removing *gfp*). The PCR fragment was cut with the same enzymes and ligated into the backbone, resulting pLS226. For the subsequent plasmids, pLS226 was linearized with EcoRV/NotI and used as backbone. *ppgA*, *pieG*, *rcc1* and *ranBP10* were amplified using oLS350/oLS216, oLS351/oLS265, oLS352/oLS353 or oLS365/oLS366, respectively, digested with EcoRV/NotI, and ligated into pLS226, resulting pLS229, pLS230, pLS231 or pLS242, respectively. For the production of GFP-tagged *L. pneumophila* effectors in mammalian cells, the corresponding genes were amplified with the primers oLS135/oLS136, oLS133/oLS134, oLS236/oLS238, oLS358/oLS359, oLS358/oLS238 or oLS363/oLS364, and inserted into pEGFP using SalI/BamHI yielding the plasmids pLS093, pLS095, pLS227, pLS233, pLS234 and pLS235, respectively.

Growth and competition of *L. pneumophila* mutant strains

Growth of *L. pneumophila* wild-type and mutant strains was analyzed in AYE broth. To this end, the bacteria were grown on CYE plates for three days and inoculated to an OD_{600} of 0.1 in AYE medium in a 96-well plate. Bacterial growth was monitored for 30 h by microtiter plate reader (Synergy H1, Biotek).

Intracellular growth of *L. pneumophila* wild-type and mutant strains in *D. discoideum,* macrophages or *A. castellanii* was assessed by determining colony forming units (CFUs). Exponentially growing *D. discoideum* or *A. castellanii* amoebae were seeded onto a 96-well plate at a density of 5×10^4 cells/well in HL5 medium (without glucose; Formedium) containing 50 mM maltose or PYG medium, respectively. RAW264.7 macrophages were seeded (1.25×10^5 cells/ml) in RPMI medium containing 10% FCS. The next day, *D. discoideum* was washed with Sörensen phosphate buffer (2 mM Na_2HPO_4, 15 mM KH_2PO_4, pH 6.0) containing 50 mM $CaCl_2$ (SorC), and further incubated until infection in MB medium (7 g of yeast extract, 14 g of thiotone E peptone, 20 mM MES, pH 6.9). Before infection, *A. castellanii* was washed with Ac buffer (4 mM $MgSO_4 \times 7\ H_2O$, 0.4 mM $CaCl_2$, 3.4 mM $Na_3C_6H_5O_7$, 0.05 mM $Na_2HPO_4 \times 7\ H_2O$, 2.5 mM KH_2PO_4, 0.05 mM NH_4Cl, 0.05 mM $FeSO_4$, pH 6.5), and further incubated and infected in this buffer . All cell types were infected (MOI 0.1) with stationary phase *L. pneumophila* grown in AYE broth and diluted in MB or RPMI medium. The infection was synchronized by centrifugation, and the infected phagocytes were incubated at 25°C (*D. discoideum*), 30°C (*A. castellanii*) or 37°C

(macrophages). At the time points indicated, the cells were lysed with 0.8% saponin (47036, Sigma-Aldrich), and serial dilutions were plated on CYE agar plates and incubated for 3 days at 37°C. CFUs were counted using a colony counter (CounterMat Flash 4000, IUL Instruments, CounterMat software).

Single round intracellular growth of GFP-producing *L. pneumophila* was assayed in *D. discoideum*. The amoebae were seeded onto a black 96-well clear bottom plate (Milian) at a density of 1.25×10^5 cells/ml. The next day, the cells were washed with LoFlo (Formedium), infected (MOI 10) for 1 h with stationary phase *L. pneumophila*, and incubated at 25°C in a fluorescence plate reader (Synergy H1, Biotek). The fluorescence was measured every 4 h for 48 h using an excitation wave length of 485 nm, and an emission of 528 nm.

For the competition assays, *A. castellanii* (2×10^4 per well, 96-well plate) in Ac buffer was co-infected (MOI 0.01) with wild-type *L. pneumophila* and the respective mutant strain. The infected amoebae were grown for 21 days at 37°C. Every third day the amoebae were lysed with 0.8% saponin. The lysate was diluted 1:1000 and used to infect fresh amoebae (50 µl lysate per well). Dilutions of the lysate were plated on CYE agar plates containing Kan (10 mg/ml) or not to determine CFU.

Translocation assay

To determine *lpg1975*-dependent translocation into host cells of LegG1 and PpgA, RAW264.7 macrophages were seeded in RPMI containing 10% FCS at a density of 2.5×10^5/ml onto 96-well plates in a final volume of 100 ml/well and incubated at 37°C/5% CO_2 overnight. The macrophages were infected with stationary phase *L. pneumophila* (MOI 20, 1 h, 37°C), grown in AYE supplemented with 0.5 mM IPTG, producing TEM β-lactamase fusion proteins. 20 ml of 6-fold CCF4/AM substrate (LiveBLAzer™ FRET-B/G Loading Kit, ThermoFisher) was added to each well 1 h p.i. After 90 min incubation in the dark, the fluorescence was measured with a fluorescence plate reader (Synergy H1, Biotek) using an excitation wave length of 405 nm and an emission of 460 nm or 535 nm. The Icm/Dot substrate LepB served as a positive control, and the cytoplasmic protein FabI as a negative control.

(Real time) fluorescence microscopy

Microscopy was performed with a Leica SP8 inverse laser scanning confocal microscope. For real-time fluorescence microscopy, exponentially growing *D. discoideum* amoebae producing calnexin-GFP were seeded in 35 mm imaging dishes (Ibidi) in HL5 medium containing G418

(20 μg/ml) to a total cell number of 4.5×10^5 cells one day prior to infection. The next day, the amoebae were infected (MOI 10) with *L. pneumophila* producing DsRed for 1 h at 25°C. At 2 h p.i. the cells were washed with LoFlo medium and left in 600 μL of the same. A 2% agar overlay was layered onto the cells, excess medium was removed and LCV motility was recorded for 200 s with 10s intervals.

To assess whether *L. pneumophila* strains affect host cell motility, "gap closure" assays were performed. *D. discoideum* producing GFP was seeded in a 6-well plate at a total cell number of 1.25×10^6/well. The next day, the amoebae were infected (MOI 5) with *L. pneumophila* producing DsRed at 25°C in HL5 medium. 1 h p.i. the cells were washed with MB medium, detached in 1.5 ml MB medium, and 70 μl of the cell suspension was seeded into each slot of a 2-well culture insert 35 mm imaging dishes (Ibidi). The cells were left to attach for 2 h, and then the inset was removed. Gap closure was recorded 0 and 3 h after removal of the inset. The profile of the GFP signal was plotted using ImageJ.

Localization of RanBP1 to the LCV was analyzed to assess the activation of Ran GTPase on this compartment. To this end, exponentially growing *D. discoideum* producing GFP-RanBP1 were infected (MOI 30) with *L. pneumophila* strains producing DsRed in T75 flasks for 1 h. Intact LCVs were purified by the two-step immuno-affinity procedure as described (Hoffmann et al., 2014; Urwyler et al., 2009). Briefly, infected amoebae were lysed in HS buffer (20 mM HEPES-KOH, pH 7.2, 250 mM sucrose, 0.5 mM EGTA) by passing the cells nine times through a stainless steel ball homogenizer (Isobiotec). Homogenates were blocked with 2% FCS, incubated with an antibody recognizing the LCV-bound effector SidC (1:3000) and subsequently with a secondary antibody coupled to magnetic beads (Miltenyi Biotec). The LCVs were enriched with a MACS separator (Miltenyi Biotec) and finally purified by a Histodenz density gradient centrifugation. Isolated LCVs were spun (500 *g*) on poly-L-lysine coated coverslips and stained with an antibody recognizing calnexin (1:200, DSHB Iowa: 270-390-2) followed by a corresponding secondary antibody (Cy5, 1:250, Jackson: 115-175-044). The samples were imaged, and the percentage of GFP-positive LCVs was counted.

Localization of *L. pneumophila* RCC1 repeat effectors was analyzed by producing GFP-fusions in *D. discoideum*. Amoebae producing GFP-PpgA, GFP-LegG1 or GFP-$LegG1_{\Delta CAAX}$ were seeded on poly-L-lysine coated coverslips one day prior to infection. The next day, the amoebae were infected (MOI 5) with *L. pneumophila* wild-type or Δ*icmT* producing DsRed for 1 h in HL5, washed with SorC buffer, fixed with 4% PFA for 30 min at RT and mounted on glass slides.

Localization of *L. pneumophila* RCC1 repeat effectors to the LCV was analyzed in homogenates of infected *D. discoideum*. Exponentially growing *D. discoideum* producing GFP-calnexin were infected (MOI 30) with *L. pneumophila* producing DsRed and V5-tagged PpgA, LegG1 or PieG for 2 h at 25°C. Homogenates were prepared as described above, fixed onto poly-L-lysine coated coverslips and stained with an antibody recognizing V5 (1:250, ThermoFisher: R960-25) and a corresponding secondary antibody coupled to Cy5 (1:250, Jackson: 115-175-044).

Imaging flow cytometry

For imaging flow cytometry (IFC) analysis of LCV markers, *D. discoideum* producing P4C-GFP or calnexin-GFP were seeded in 12-well plates (5×10^5 cells per well) one day prior to infection. The following day, the amoebae were infected (MOI 5) with *L. pneumophila* strains producing DsRed in HL5. At the indicated time points, the amoebae were detached from the surface, washed with SorC buffer and fixed in solution with 4% PFA for 30 min at 4°C. After washing, the cells were resuspended in PBS and analyzed by IFC (ImageStreamX MkII; Amnis) as previously described (Welin et al., 2018). Briefly, 10,000 cells were acquired, and after color compensation, analysis of the bright detail similarity between GFP (P4C or calnexin) and DsRed (*L. pneumophila*) was carried out in single GFP-positive cells containing one LCV (generally >1000 cells per sample), using the IDEAS 6.2 software (Amnis). The resulting IFC score represents the degree of colocalization between DsRed and GFP, and thus accumulation of P4C or calnexin on the LCV.

Yeast methods

Localization of *L. pneumophila* proteins in yeast was analyzed in exponentially growing yeast producing GFP fusion proteins under control of the *gal* promoter. The yeast wild-type strain BY4741 was grown overnight to saturation in SG-Leu (ForMedium), diluted, and incubated for 3 h at 30°C. The cells were washed with H_2O, mounted onto glass slides and imaged with a Leica SP8 al microscope.

Spot assays were performed to assess whether overexpression of *L. pneumophila* RCC1 repeat effectors affects yeast growth. To this end, the yeast wild-type or mutant strains producing FLAG fusion proteins of *L. pneumophila* effectors under control of the *gal* promoter were grown to logarithmic phase, and 9 µl of tenfold dilutions were spotted on SG-Leu (ForMedium) plates and incubated for 3-7 days at the indicated temperatures. Yeast growth was documented by camera, and colony size at 20°C was measured in ImageJ.

To monitor the growth of yeast in broth, the yeast wild-type strain or *rna1-1* mutant strain producing *L. pneumophila* effectors were grown on SG-Leu plates and inoculated in 96-well plates to an OD_{600} of 0.1 in SG-Leu medium (ForMedium). Yeast growth was measured by OD_{600} in a fluorescence plate reader (Synergy H1, Biotek) for one week at 20°C.

To validate the interaction between LegG1 and potential interaction partners identified by mass spectroscopy, plasmids producing LegG1 fused at its N-terminus to *GAL4-AD* (activation domain), and RanBP10, RanGAP or Ran, wild-type and mutants, fused at their N-terminus to *GAL4-DBD* (DNA binding domain) were transformed into the yeast two-hybrid strain AH109. The transformants were grown on SD-Leu-Trp plates (ForMedium), inoculated in H_2O and spotted in tenfold dilution series on SG-His or SD-Leu-Trp plates (ForMedium). The plates were incubated for 4-6 days at 30°C, and yeast growth was documented by camera. In this assay, the physical interaction between two proteins results in the assembly of the transcription factor and activation of the HIS3 reporter gene. Presence of the plasmids is controlled by growth on SD-plates without leucine and tryptophan. To confirm the interaction between LegG1 and RanBP10, a yeast two-hybrid experiment was performed using plasmids producing LegG1, PpgA or PieG fused at their N-terminus to *GAL4-AD* (activation domain), and RanBP10 at its N-terminus to *GAL4-DBD* (DNA binding domain).

Co-immunoprecipitations

Affinity purification of FLAG-tagged proteins from yeast was performed to identify interaction partners of *L. pneumophila* RCC1 repeat effectors. FLAG-tagged *L. pneumophila* effectors were co-produced with Strep-tagged proteins in the yeast wild-type strain BY4741 under control of the *gal* promoter. The cells were grown to an OD_{600} 2.0-3.0, lysed in yeast lysis buffer (25 mM HEPES pH 8.0, 150 mM KCl, 2 mM $MgCl_2$, 0.1 mM EDTA, 0.5 mM EGTA, 15% glycerol, 0.1% NP40, Roche complete protease inhibitor) using a Pulverisette mill. FLAG fusions and associated Strep fusions were pulled down from cell lysates using anti-FLAG M2 affinity gel (Sigma: A2220) according to the manufacturer's protocol. Briefly, lysate supernatant was incubated with affinity beads for 2 h at 4°C in rotation. Beads were washed three times with lysis buffer, resuspended in 2× SDS loading buffer and analyzed by Western blot using antibodies (4°C, overnight) against Strep-tag (1:1000, Abcam: ab76949) or FLAG-tag (1:1000, ThermoFisher: MAI-91878), and the corresponding secondary antibodies coupled to HRPO (1:1000, GE Healthcare: NA934 or NA931, respectively) using PBS/2% milk as a blocking reagent, followed by detection by chemiluminescence (Amersham ECL kit; GE Healthcare: RPN2109).

For the analysis of PieG binding to endogenous GSP1 (Ran), FLAG-tagged *L. pneumophila* effectors were produced in the yeast wild-type strain BY4741. Cells were processed as above and analyzed by Western blot upon immunostaining (4°C, overnight) for GSP1 or FLAG-tag (1:1000, ThermoFisher: MAI-91878) and with the corresponding secondary antibody coupled to HRPO (1:1000, GE Healthcare: NA934 or NA931, respectively) using 2% milk as a blocking reagent, followed by detection by chemiluminescence (Amersham ECL kit; GE Healthcare: RPN2109).

Strep-tagged $LegG1_{33\text{-}286}$ was purified from *E. coli* using a pET51b-based expression vector (pM1319). Strep-tagged $LegG1_{33\text{-}286}$ was pre-incubated with HEK 293T lysate for 3 h at 4°C before Strep-$LegG1_{33\text{-}286}$ was coupled to a Strep-Tactin Sepharose (IBA) for 2 h at 4°C. To remove unspecific bound proteins, the resin was washed 3 times before Strep-$LegG1_{33\text{-}286}$ together with its bound interaction partners was eluted by 10 mM desthiobiotin. Elution fractions from the LegG1-pulldown and a control pulldown without LegG1 were TCA-precipitated and separated by SDS-PAGE. Additional protein bands were visualized by blue-silver staining, cut out and analyzed by mass spectrometry.

To confirm the interaction between LegG1 and RanBP10 observed by yeast two-hybrid, HEK 293T cells were co-transfected using lipofectamine 3000 (Invitrogen) reagent with plasmids producing GFP-fusions of Lpg1975, LegG1, Lpg1975-LegG1_fusion, PpgA, PieG or $PieG_{190\text{-}475}$ and Strep-tagged RanBP10. 24 h post transfection the cells were collected, washed twice with ice-cold dPBS and lysed 1 h at 4°C in 1 ml 1× lysis buffer (25 mM HEPES pH 8.0, 150 mM KCl, 2 mM $MgCl_2$, 0.1 mM EDTA, 0.5 mM EGTA, 15% glycerol, 0.1% NP40, Roche complete protease inhibitor). The lysate was cleared by centrifugation for 10 min, 14 000 *g* at 4°C and the supernatant was collected and incubated overnight with 3µl antibody against Strep-tag (1:1000, Abcam: ab76949) while rotating at 4°C. The subsequent day, 40 µl washed A/G agarose beads (Pierce) were added and the sample was further incubated for 2 h on the wheel at 4°C. Afterward, beads were washed three times with lysis buffer, resuspended in 2× NuPAGE™ LDS Sample Buffer (ThermoFisher) and analyzed by Western blot using antibodies (4°C, overnight) against GFP-tag (1:1000, Clontech: 632380) or Strep-tag (1:1000, Abcam: ab76949), and the corresponding secondary antibodies coupled to HRPO (1:1000, GE Healthcare: NA931 or NA934, respectively) using TBS/3%BSA or TBST/2% milk, respectively, as a blocking reagent, followed by detection by chemiluminescence (Amersham ECL kit; GE Healthcare: RPN2109).

Mass spectrometry analysis

For the identification of LegG1 in *L. pneumophila* lysates, 40 µg of protein were diluted in 50 µL of 10 mM Tris/2 mM $CaCl_2$ buffer (pH 8.2) and enzymatically digested using 5 µLof trypsin (100 ng/µL in 10 mM HCl). Digestion was carried out in a microwave instrument (Discover System, CEM) for 30 min at 5 W and 60°C. Samples were dried in a SpeedVac (Savant). For LC-MS/MS analysis, the samples were dissolved in 0.1% formic acid (Romil), diluted 1:10 and analyzed on a nanoAcquity UPLC (Waters Inc.) connected to a Q Exactive mass spectrometer (Thermo Scientific) equipped with a Digital PicoView source (New Objective). Peptides were trapped on a Symmetry C18 trap column (5 µm, 180 µm × 20 mm, Waters Inc.) and separated on a BEH300 C18 column (1.7 µm, 75 µm × 150 m, Waters Inc.) at a flow rate of 250 nL/min using a gradient from 1% solvent B (0.1% formic acid in acetonitrile, Romil)/99% solvent A (0.1% formic acid in water, Romil) to 40% solvent B/60% solvent A within 90 min. The mass spectrometer was operated in data-dependent mode (DDA), acquiring a full-scan MS spectra (350-1500 m/z) at a resolution of 70000 at 200 m/z after accumulation to a target value of 3000000, followed by HCD (higher-energy collision dissociation) fragmentation on the twelve most intense signals per cycle. HCD spectra were acquired at a resolution of 35000 using a normalized collision energy of 25 and a maximum injection time of 120 ms. The automatic gain control (AGC) was set to 50000 ions. Charge state screening was enabled and singly and unassigned charge states were rejected. Only precursors with intensity above 8300 were selected for MS/MS (2% underfill ratio). Precursor masses previously selected for MS/MS measurement were excluded from further selection for 30 s, and the exclusion window was set at 10 ppm. The samples were acquired using internal lock mass calibration on m/z 371.1010 and 445.1200. Proteins were identified using the PEAKS search engine (PEAKS X, Bioinformatic Solutions) performing a de-novo search and an identification against a Uniprot database containing *L. pneumophila* subsp. pneumophila (strain Philadelphia 1 / ATCC 33152 / DSM 7513, v. 2019-07-29). Data were searched with a fragment ion mass tolerance of 0.02 Da and a parent ion tolerance of 15.0 PPM. Oxidation of methionine was specified in Mascot as a variable modification. Peptide identifications were accepted if they achieved a peptide false discovery rate (FDR) of less than 0.1%, and the proteins contained at least 2 identified peptides (Table S2C).

For the identification of protein binding partners of *L. pneumophila* RCC1 repeat effector, tryptic peptides were separated and analyzed by nano-HPLC/MS/MS. The separations were carried out on an UltiMateTM 3000 RSLCnano system (Dionex, Germany). The MS and MS/MS experiments were carried out on a Q Exactive Plus^{TM} Hybrid Quadrupole-Orbitrap

Mass Spectrometer equipped with a nano-spray source (Nanospray Flex Ion Source, Thermo Scientific). All solvents were LC-MS grade. The lyophilized tryptic peptides were dissolved in 20 µL 0.1% TFA in water. 3 µL of sample were injected onto a pre-column cartridge (5 µm, 100 Å, 300 µm ID × 5 mm; Dionex, Germany) using 0.1 % TFA in water as eluent with a flow rate of 30 µL/min. Desalting was performed for 5 min with eluent flow to waste, followed by back-flushing of the sample during the whole analysis from the pre-column to the PepMap100 RSLC C18 nano-HPLC column (2 µm, 100 Å, 75 µm ID × 50 cm, nanoViper; Dionex, Germany). The following linear gradient was used for peptide separation: starting conditions 95% solvent A / 5% solvent B, linear increase to 70% A / 30% B in 90 min, further linear increase to 40% A / 60% B in 5 min, further linear increase to 5% A / 95% B in 5 min, washing with these conditions for 5 min, back-flushing and re-equilibration to starting conditions. Water containing 0.1% formic acid was used as solvent A, acetonitrile containing 0.1% formic acid as solvent B. The nano-HPLC was online coupled to the Quadrupole-Orbitrap Mass Spectrometer using a standard coated SilicaTip (ID 20 µm, Tip-ID 10 µM, New Objective, Woburn, MA, USA). Mass range of m/z 300 to 1650 was acquired with a resolution of 70000 for full scan, followed by up to ten high energy collision dissociation (HCD) MS / MS scans of the most intense at least doubly charged ions.

Data evaluation was performed using MaxQuant software (Cox and Mann, 2008) (v.1.5.3.30) including the Andromeda search algorithm and searching in parallel the human, *E. coli*, and *L. pneumophila* reference proteome of the Uniprot database. The search was performed for full enzymatic trypsin cleavages allowing two miscleavages. For protein modifications carbamidomethylation was chosen as fixed and oxidation of methionine and acetylation of the N-terminus as variable modifications. The mass accuracy for full mass spectra was set to 4.5 ppm and for MS/MS spectra to 20 ppm. The false discovery rates for peptide and protein identification were set to 1 %. Only human proteins for which at least two peptides were quantified were chosen for further validation. Relative quantification of proteins was carried out using the label-free quantification algorithm implemented in MaxQuant. Further data evaluation was performed using Perseus software (Tyanova and Cox, 2018) (v. 1.5.2.6). Proteins not identified with at least two peptides in at least one of the samples and known contaminations were filtered off. Samples resulting from pulldowns using the active or inactive probe, respectively, were grouped together. Label-free quantification (LFQ) intensities were logarithmized (log2), and proteins, which were not three times quantified in at least one of the groups, were filtered off. Missing values were imputed using small normal distributed values (width 0.3, down shift 2.3) and a two sided t-

test (s0 = 1, FDR 0.01) was performed. Statistically significant proteins enriched by the active compared to the inactive probe were considered as hits (see Table S2B and Table S2C for a list of identified proteins and their statistical significance).

Ran activation assay

The effect of *Legionella* RCC-repeat proteins on the cellular amount of Ran(GTP) was analyzed using a 'Ran Activation Assay Kit' (Cell Biolabs). HEK 293T cells were cultivated in T75 flasks and transfected with pEGFP plasmids encoding Strep-fusion proteins using lipofectamine 3000 (Invitrogen). After 24 h, the cells were harvested with Trypsin-EDTA, washed with PBS and lysed for 20 min on ice in 1 ml 1× lysis buffer (containing protease inhibitor cocktail, Sigma). The samples were centrifuged for 10 min at 14 000 *g* at 4°C and the supernatant was transferred to a fresh tube. A small fraction was kept for the analysis of the total amount of Ran by Western blot. For the positive and negative controls, a sample was split in two and loaded with GTPγS or GDP, respectively, for 30 min at 30°C in presence of 10 mM EDTA. The loading was stopped by addition of 60 mM $MgCl_2$. Total protein concentration was measured by NanoDrop (ThermoFisher) and 40 µl RanBP1 PBD agarose bead slurry was added to 1 mg sample and incubated for 1 h at 4°C on a wheel. Beads were pelleted by centrifugation for 10 sec at 14 000 *g* at 4°C, washed three times with 500 µl 1× lysis buffer and resuspended in 40 µl 2× NuPAGE™ LDS Sample Buffer (ThermoFisher). Samples were incubated for 5 min at 70 °C, spun and the supernatant was analyzed by Western blot. After Western blotting, nitrocellulose membranes were immunostained (2 h, RT) for Ran (1:1000, 240902, Cell biolabs) or GAPDH (1:1000, 2118, Cell Signaling Technology) and with the corresponding secondary antibody linked to HRPO (GE Healthcare Life Sciences) using TBST/5% milk (Roth) as a blocking reagent. After extensive washing, the enhanced chemiluminescence (ECL) signal was detected with an ImageQuant LAS4000 (GE Healthcare Life Sciences). Signal intensities were quantified with ImageQuant TL (GE Healthcare Life Sciences).

RNA interference and determination of protein depletion efficiency

For the RNA interference experiments, A549 cells were grown in 96-well plates and treated for 48 h with a final concentration of 10 nM of siRNA oligonucleotides (Table S1C). To this end, the siRNA stock (10 µM) was diluted 1:15 in RNAse-free water, and 3 µl of diluted siRNA was added per well. Allstars siRNA (Qiagen) was used as a negative control. Subsequently, 24.25 µl RPMI medium without FBS was mixed with 0.75 µl HiPerFect

transfection reagent (Qiagen), added to the well, mixed, and incubated for 5-10 min at room temperature. In the meantime, cells were diluted in suppl. RPMI medium, and 175 µl of the diluted cells (2×10^4 cells) was added on top of each siRNA-HiPerFect transfection complex and incubated for 48 h. The cells were infected with GFP-producing *L. pneumophila* strains, and intracellular replication was determined by fluorescence as described above.

The protein depletion efficiency was assessed by Western blot. A549 cells were grown in 24-well plates and treated for 48 h with a final concentration of 10 nM of siRNA oligonucleotides (Table S1C). To this end, the siRNA stock (10 µM) was diluted 1:15 in RNAse-free water, and 9 µl of diluted siRNA was added per well. Allstars siRNA (Qiagen) was used as a negative control. Subsequently, 72.75 µl RPMI medium without FBS was mixed with 2.25 µl HiPerFect transfection reagent (Qiagen), added to the well, mixed, and incubated for 5-10 min at RT. In the meantime, cells were diluted in suppl. RPMI medium, and 525 µl of the diluted cells (6×10^4 cells) was added on top of each siRNA-HiPerFect transfection complex and incubated for 48 h. Protein depletion efficiency was assessed as follows: cells were harvested in ice-cold PBS and lysed with ice-cold NP-40 cell lysis buffer, and cell extracts were subjected to SDS–PAGE. After Western blotting, nitrocellulose membranes were immunostained (2 h, RT) for RanGAP (1:500, ab92360, Abcam), RanBP10 (1:1000, ab235104, Abcam) or GAPDH (1:1000, 2118, Cell Signaling Technology) and with the corresponding secondary antibody linked to HRPO (GE Healthcare Life Sciences) using TBST/5% milk (Roth) as a blocking reagent. After extensive washing, the enhanced chemiluminescence (ECL) signal was detected with an ImageQuant LAS4000 (GE Healthcare Life Sciences).

To assess cell viability after siRNA treatment, propidium iodide (PI) uptake was measured. A549 cells were cultured and treated with siRNA oligonucleotides (Table S1C) as described above (protein depletion efficiency). The cells were then harvested in ice-cold PBS, stained for 1-2 min with 1.5 µg/ml PI (Life Technologies) in 500 µl PBS and subjected to flow cytometry analysis (Attune Nxt Acousting Focusing Cytometer, Invitrogen). Gates were set according to forward/sideward scatter properties, and 100,000 events were collected for each sample with the Attune Nxt Software v.2.7.0. Cells treated for 10 min with 70% sterile-filtered ethanol (EtOH) served as positive control for cell death. PI (Y1-channel) was determined on single cells. 70% EtOH cells account as 100% PI-uptake and % of other cells was calculated by dividing PI-positive cells by the average EtOH-PI-positive cells.

References

Alexeyev, M.F., Shokolenko, I.N., and Croughan, T.P. (1995). Improved antibiotic-resistance gene cassettes and omega elements for *Escherichia coli* vector construction and *in vitro* deletion/insertion mutagenesis. Gene *160*, 63-67.

Azuma, Y., Seino, H., Seki, T., Uzawa, S., Klebe, C., Ohba, T., Wittinghofer, A., Hayashi, N., and Nishimoto, T. (1996). Conserved histidine residues of RCC1 are essential for nucleotide exchange on Ran. J Biochem *120*, 82-91.

Bärlocher, K., Hutter, C.A.J., Swart, A.L., Steiner, B., Welin, A., Hohl, M., Letourneur, F., Seeger, M.A., and Hilbi, H. (2017). Structural insights into *Legionella* RidL-Vps29 retromer subunit interaction reveal displacement of the regulator TBC1D5. Nat Commun *8*, 1543.

Cazalet, C., Rusniok, C., Brüggemann, H., Zidane, N., Magnier, A., Ma, L., Tichit, M., Jarraud, S., Bouchier, C., Vandenesch, F*., et al.* (2004). Evidence in the *Legionella pneumophila* genome for exploitation of host cell functions and high genome plasticity. Nat Genet *36*, 1165-1173.

Chen, J., de Felipe, K.S., Clarke, M., Lu, H., Anderson, O.R., Segal, G., and Shuman, H.A. (2004). *Legionella* effectors that promote nonlytic release from protozoa. Science *303*, 1358-1361.

Cox, J., and Mann, M. (2008). MaxQuant enables high peptide identification rates, individualized p.p.b.-range mass accuracies and proteome-wide protein quantification. Nat Biotechnol *26*, 1367-1372.

de Felipe, K.S., Glover, R.T., Charpentier, X., Anderson, O.R., Reyes, M., Pericone, C.D., and Shuman, H.A. (2008). *Legionella* eukaryotic-like type IV substrates interfere with organelle trafficking. PLoS Pathog *4*, e1000117.

Edgar, R.C. (2004). MUSCLE: multiple sequence alignment with high accuracy and high throughput. Nucleic Acids Res *32*, 1792-1797.

Finsel, I., Ragaz, C., Hoffmann, C., Harrison, C.F., Weber, S., van Rahden, V.A., Johannes, L., and Hilbi, H. (2013). The *Legionella* effector RidL inhibits retrograde trafficking to promote intracellular replication. Cell Host Microbe *14*, 38-50.

Hoffmann, C., Finsel, I., Otto, A., Pfaffinger, G., Rothmeier, E., Hecker, M., Becher, D., and Hilbi, H. (2014). Functional analysis of novel Rab GTPases identified in the proteome of purified *Legionella*-containing vacuoles from macrophages. Cell Microbiol *16*, 1034-52.

Künzler, M., Trueheart, J., Sette, C., Hurt, E., and Thorner, J. (2001). Mutations in the YRB1 gene encoding yeast ran-binding-protein-1 that impair nucleocytoplasmic transport and suppress yeast mating defects. Genetics *157*, 1089-1105.

Letunic, I., and Bork, P. (2007). Interactive Tree Of Life (iTOL): an online tool for phylogenetic tree display and annotation. Bioinformatics *23*, 127-128.

Li, L., Stoeckert, C.J., Jr., and Roos, D.S. (2003). OrthoMCL: identification of ortholog groups for eukaryotic genomes. Genome research *13*, 2178-2189.

Loovers, H.M., Kortholt, A., de Groote, H., Whitty, L., Nussbaum, R.L., and van Haastert, P.J. (2007). Regulation of phagocytosis in *Dictyostelium* by the inositol 5-phosphatase OCRL homolog Dd5P4. Traffic *8*, 618-628.

Mampel, J., Spirig, T., Weber, S.S., Haagensen, J.A.J., Molin, S., and Hilbi, H. (2006). Planktonic replication is essential for biofilm formation by *Legionella pneumophila* in a complex medium under static and dynamic flow conditions. Appl Environ Microbiol *72*, 2885-2895.

Mitchell, A., Chang, H.Y., Daugherty, L., Fraser, M., Hunter, S., Lopez, R., McAnulla, C., McMenamin, C., Nuka, G., Pesseat, S*., et al.* (2015). The InterPro protein families database: the classification resource after 15 years. Nucleic Acids Res *43*, D213-221.

Müller-Taubenberger, A., Lupas, A.N., Li, H., Ecke, M., Simmeth, E., and Gerisch, G. (2001). Calreticulin and calnexin in the endoplasmic reticulum are important for phagocytosis. EMBO J *20*, 6772-6782.

Renault, L., Nassar, N., Vetter, I., Becker, J., Klebe, C., Roth, M., and Wittinghofer, A. (1998). The 1.7 A crystal structure of the regulator of chromosome condensation (RCC1) reveals a seven-bladed propeller. Nature *392*, 97-101.

Rothmeier, E., Pfaffinger, G., Hoffmann, C., Harrison, C.F., Grabmayr, H., Repnik, U., Hannemann, M., Wölke, S., Bausch, A., Griffiths, G*., et al.* (2013). Activation of Ran GTPase by a *Legionella* effector promotes microtubule polymerization, pathogen vacuole motility and infection. PLoS Pathog *9*, e1003598.

Sadosky, A.B., Wiater, L.A., and Shuman, H.A. (1993). Identification of *Legionella pneumophila* genes required for growth within and killing of human macrophages. Infect Immun *61*, 5361-5373.

Schlenstedt, G., Smirnova, E., Deane, R., Solsbacher, J., Kutay, U., Görlich, D., Ponstingl, H., and Bischoff, F.R. (1997). Yrb4p, a yeast ran-GTP-binding protein involved in import of ribosomal protein L25 into the nucleus. EMBO J *16*, 6237-6249.

Segal, G., and Shuman, H.A. (1998). Intracellular multiplication and human macrophage killing by *Legionella pneumophila* are inhibited by conjugal components of IncQ plasmid RSF1010. Mol Microbiol *30*, 197-208.

Tiaden, A., Spirig, T., Weber, S.S., Brüggemann, H., Bosshard, R., Buchrieser, C., and Hilbi, H. (2007). The *Legionella pneumophila* response regulator LqsR promotes host cell

interactions as an element of the virulence regulatory network controlled by RpoS and LetA. Cell Microbiol *9*, 2903-2920.

Treangen, T.J., Ondov, B.D., Koren, S., and Phillippy, A.M. (2014). The Harvest suite for rapid core-genome alignment and visualization of thousands of intraspecific microbial genomes. Genome Biol *15*, 524.

Tyanova, S., and Cox, J. (2018). Perseus: a bioinformatics platform for integrative analysis of proteomics data in cancer research. Methods Mol Biol *1711*, 133-148.

Urwyler, S., Nyfeler, Y., Ragaz, C., Lee, H., Mueller, L.N., Aebersold, R., and Hilbi, H. (2009). Proteome analysis of *Legionella* vacuoles purified by magnetic immunoseparation reveals secretory and endosomal GTPases. Traffic *10*, 76-87.

Veltman, D.M., Akar, G., Bosgraaf, L., and Van Haastert, P.J.M. (2009). A new set of small, extrachromosomal expression vectors for *Dictyostelium discoideum*. Plasmid *61*, 110-118.

Waterhouse, A., Bertoni, M., Bienert, S., Studer, G., Tauriello, G., Gumienny, R., Heer, F.T., de Beer, T.A.P., Rempfer, C., Bordoli, L*., et al.* (2018). SWISS-MODEL: homology modelling of protein structures and complexes. Nucleic Acids Research *46*, W296-W303.

Weber, S., and Hilbi, H. (2014). Live cell imaging of phosphoinositide dynamics during *Legionella* infection. Methods Mol Biol *1197*, 153-167.

Weber, S., Wagner, M., and Hilbi, H. (2014). Live-cell imaging of phosphoinositide dynamics and membrane architecture during *Legionella* infection. mBio *5*, e00839-13.

Weber, S.S., Ragaz, C., and Hilbi, H. (2009). The inositol polyphosphate 5-phosphatase OCRL1 restricts intracellular growth of *Legionella*, localizes to the replicative vacuole and binds to the bacterial effector LpnE. Cell Microbiol *11*, 442-460.

Welin, A., Weber, S., and Hilbi, H. (2018). Quantitative imaging flow cytometry of *Legionella*-infected *Dictyostelium* amoebae reveals the impact of retrograde trafficking on pathogen vacuole composition. Appl Environ Microbiol *84*, e00158-18.

Wiater, L.A., Sadosky, A.B., and Shuman, H.A. (1994). Mutagenesis of *Legionella pneumophila* using Tn*903*dll*lacZ*: identification of a growth-phase-regulated pigmentation gene. Mol Microbiol *11*, 641-653.

Yan, C., Lee, L.H., and Davis, L.I. (1998). Crm1p mediates regulated nuclear export of a yeast AP-1-like transcription factor. EMBO J *17*, 7416-7429.
